# Enhancing breath-based diagnostics through eXplainable Artificial Intelligence

**DOI:** 10.1371/journal.pone.0351833

**Published:** 2026-06-26

**Authors:** Andrea Lo Sasso, Nicola Amoroso, Domenico Diacono, Marianna La Rocca, Alfonso Monaco, Ester Pantaleo, Sabina Tangaro, Loredana Bellantuono, Roberto Bellotti

**Affiliations:** 1 Dipartimento Interuniversitario di Fisica “M. Merlin”, Università degli Studi di Bari Aldo Moro, Bari, Italy; 2 Istituto Nazionale di Fisica Nucleare (INFN), Sezione di Bari, Bari, Italy; 3 Predict S.r.l, Fiera del Levante, Bari, Bari, Italy; 4 Dipartimento di Farmacia - Scienze del Farmaco, Università degli Studi di Bari Aldo Moro, Bari, Italy; 5 Dipartimento di Scienze del Suolo, della Pianta e degli Alimenti, Università degli Studi di Bari Aldo Moro, Bari, Italy; 6 Dipartimento di Biomedicina Traslazionale e Neuroscienze (DiBraiN), Università degli Studi di Bari Aldo Moro, Bari, Italy; Korea University - Seoul Campus: Korea University, KOREA, REPUBLIC OF

## Abstract

Breath analysis is emerging as a non-invasive and promising diagnostic approach capable of assessing a patient’s metabolic state by detecting volatile organic compounds in exhaled breath. This study investigates the potential of breath analysis for the early detection of lung cancer, respiratory and gastrointestinal diseases using open-access data from three distinct datasets. An artificial intelligence methodology is implemented to predict diagnostic labels while addressing class imbalance, an inherent challenge in medical datasets. After evaluating model performance and stability, the most relevant volatile organic compounds identified by the best-performing model for each dataset are analyzed. Using eXplainable Artificial Intelligence, the influence of volatile organic compound abundances on predictions is examined, enabling the identification of key variables and improving model interpretability. The proposed methodology provides a robust framework for breath-based diagnostics, emphasizing the potential of integrating breath analysis with machine learning to advance clinical decision-making despite ongoing challenges related to sampling variability, detection sensitivity, and standardization across studies.

## Introduction

Scientific progress and technological advancements have paved the way for innovative and promising medical approaches. One standout example is breath analysis, which now leverages a range of advanced technologies, including spectrometry, spectroscopy, and electronic noses [1]. Whether used individually or in combination, these technologies have demonstrated potential in experimental settings to detect diseases early, monitor patient health, and offer non-invasive, efficient alternatives to traditional diagnostic and monitoring methods.

Breath analysis works by detecting and measuring volatile organic compounds (VOCs) and other gases in a person’s exhaled breath, which can serve as biomarkers for various diseases and health conditions. These compounds are released into the breath through metabolic processes in the body, and their presence or concentration can indicate specific biological changes, including the onset of disease [2,3].

VOC analysis has shown promising applications in the diagnosis of lung cancer [4]. For instance, Rai et al. demonstrated that seven key VOCs in exhaled breath could differentiate lung cancer patients from healthy controls and those with benign pulmonary nodules, achieving a mean accuracy of 92% [5]. This outcome underscores the potential of breath analysis as a non-invasive diagnostic tool that can quantitatively assess VOC profiles. Monitoring respiratory diseases like asthma and COPD is also among the most impactful breath analysis applications, enabling better management and early intervention for these chronic conditions [1]. Breath analysis also shows promise for detecting other lung diseases, airway inflammation, gastrointestinal disorders, metabolic disorders, and kidney disease [6]. For instance, the analysis of VOC patterns in the breath of pediatric patients with Inflammatory Bowel Disease (IBD) revealed that specific VOCs could effectively distinguish IBD patients from healthy controls [7]. Breath analysis has shown great promise in early disease detection and monitoring across a range of conditions. This potential was further accelerated by the COVID-19 pandemic, which highlighted the urgent need for rapid, non-invasive, and cost-effective alternatives to traditional diagnostic methods. As a result, significant progress has been made in developing breath analysis for diagnostic and screening purposes, not only for respiratory diseases like asthma and COPD but also for detecting COVID-19 and other infections. In the long term, breath analysis could become a routine method for diagnosing and monitoring respiratory illnesses and their long-term effects, as well as identifying a wide range of other diseases.

Breath analysis is already widely adopted to perform some standard analyses. For example, lactose intolerance can be commonly detected by monitoring the body’s response and measuring hydrogen levels in the breath [8]. Moreover, there is significant interest in the vast amount of information that breath analysis can provide, much of which remains unexplored.

Nowadays, thanks to the ever-increasing amount of data shared within the scientific community, it is possible to train Artificial Intelligence (AI) models that serve as supportive tools for analysis, representing a significant instrument for innovation. Machine Learning (ML) algorithms, a subset of models in the AI domain, are rapidly transforming medical physics and healthcare, demonstrating superior performance compared to conventional statistical methods in various tasks, such as signal analysis and decision-making [9], assisting in predictions related to gene expression [10,11], environmental factors [12], and cancer diagnosis [13–15]. These tools support physicians, especially in the diagnosis of controversial cases, by providing additional information that ensures more informed decision-making. Given AI’s critical role, fundamental requisites to boost its adoption in clinical practice are transparency and interpretability of the prediction process and outcomes. In the case of classification based on VOCs, it is crucial to identify which compounds impact the predictions returned by AI algorithms the most. Another challenge is the class imbalance of clinical datasets, characterized, e.g., by a disproportion between the number of healthy controls and individuals affected by a given disease. Class imbalance hinders the training of ML algorithms, making them suboptimal for diagnostics [16].

**Fig 1:**
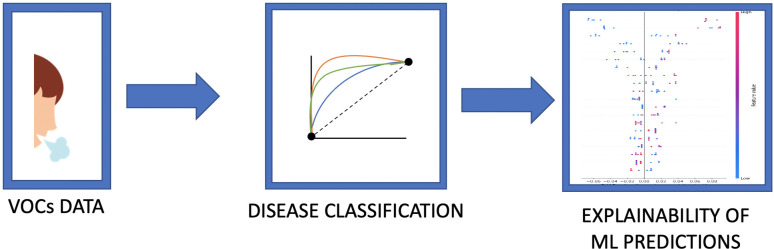
Overview of the general workflow used in this study.

To achieve robust predictions even in the presence of imbalanced datasets, we implement an ML-based methodology to classify the labels of various diseases based on VOC analysis; a workflow containing the key steps of this procedure is presented in Fig 1. We obtain data from three publicly available repositories consisting of: (i) patients with lung cancer and comparative controls [5], (ii) patients with asthma, bronchiectasis, and Chronic Obstructive Pulmonary Disease [17], (iii) pediatric patients with Inflammatory Bowel Disease and controls [7]. For each dataset, we test several ML classifiers and identify the best performing one based on its AUC score [18]. As detailed in the following, our workflow is optimized to predict the aforementioned diseases, minimizing class imbalance in each dataset. Additionally, we estimate the impact of features on the outcomes of ML models using an eXplainable Artificial Intelligence (XAI) analysis.

This article begins with the Materials and methods section, where we describe the datasets and the workflow, which consists of ML and XAI algorithms. Then, in the Results section, we report the performances of ML models and the VOCs with the highest impact on their predictions; in the Discussion, we compare our results with the state of the art and highlight further research perspectives of adopting breath analysis for the diagnosis of the considered pathologies.

## Materials and methods

### Sources of data

In this study, we employ three datasets of VOC abundances in breath samples. The first dataset [5], referred to as *Lung Cancer*, includes 427 subjects, of which 193 are labeled as healthy controls (both smokers and non-smokers), 65 are labeled as patients with benign pulmonary nodules, and 153 are labeled as patients with lung cancer. Healthy controls were recruited primarily from the family members of patients, provided they were free of chronic lung disease. Clinical diagnoses were rigorously confirmed via gold-standard methods, including CT and PET scans or histopathological analysis, prioritizing the identification of primary oncological pathologies. In this study, abundances of 28 VOCs have been obtained using silicon microreactor technology. The second dataset [17], henceforth called *Respiratory Diseases*, records VOCs from the breath of 121 individuals: 33 with Chronic Obstructive Pulmonary Disease (COPD), 35 with bronchiectasis, and 53 with asthma; for each of them, data on 70 VOCs have been collected employing GC‒MS analysis. The third dataset [7], known as *Inflammatory Bowel Disease (IBD)*, consists of 200 VOCs collected from the alveolar breath of children aged between 10 and 17 years, using ion molecule reaction mass spectrometry. The cohort includes 234 subjects, of which 167 are labeled as surgical controls with no evidence of gastrointestinal issues, 34 as patients with Crohn’s disease, and 33 as patients with Ulcerative Colitis. The control group was selected from patients hospitalized with non-gastrointestinal issues (e.g., orthopedic or dental surgery) and carefully evaluated to exclude any gastrointestinal symptoms or pre-existing chronic conditions. To provide a comprehensive overview of the data analyzed in this study, Table 1 summarizes the three datasets, detailing the individual classes, their respective sample sizes, and the number of VOC features utilized for each study. The complete lists of VOCs identified for each dataset are provided in Supplementary S2 File, while the VOCs shared across the different datasets considered in this work are detailed in the Additional Data Information section of the Supplementary Information S1 File. In this work, no dimensionality reduction techniques were applied to the VOC datasets. While the VOCs were extracted and pre-processed within the three original, publicly shared studies [5,7,17], no further treatment or dimensionality reduction was performed in the present work.

**Table 1:** Overview of the datasets analyzed in this study, detailing the individual classes, age, sex, their respective sample sizes, and the number of VOC features utilized for each study. In the Sex column, females and males are indicated with F and M, respectively. Age is expressed in years as median and interquartile range in parenthesis.

Dataset	Class label	Size	Sex (F/M)	Age (range)	Total	VOCs
Lung Cancer	Lung Cancer	153	81 / 75	65 (58–72)		
	Benign Nodules	65	32 / 33	54 (45–63)	427	28
	Healthy Controls	193	108 / 86	49 (38–61)		
Respiratory Diseases	Asthma	53	32 / 21	51 (38-66)		
	Bronchiectasis	35	24 / 11	60 (52-66)	121	70
	COPD	33	5 / 28	69 (62-73)		
Inflammatory Bowel Disease (IBD)	Ulcerative Colitis (UC)	33	15 / 18	14 (12-16)		
	Crohn’s Disease (CD)	34	16 / 18	15 (14-16)	234	200
	Healthy Controls	167	72 / 95	13 (11-14)		

### Machine learning workflow

**Fig 2:**
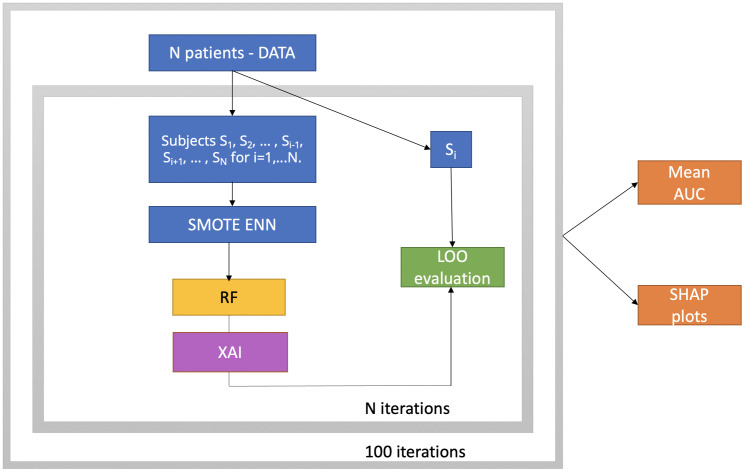
Overview of the Machine Learning workflow adopted in the present study. We implement a leave-one-out (LOO) cross-validation approach and address class imbalance using a customized SMOTE-ENN framework. The algorithm is applied in either one or two iterations, depending on the dataset’s intrinsic characteristics and the resulting class distribution after the initial cleaning step, to ensure a balanced and noise-free training set without generating spurious patterns. Then, we use the Random Forest (RF) algorithm to perform classification and the XAI analysis to quantify the impact of VOCs on predictions. For further details, refer to paragraphs SMOTE-ENN and eXplainable Artificial Intelligence (XAI).

The goal of the present study is twofold: (1) evaluating the predictive power of the VOCs in distinguishing different diseases and (2) identifying the most predictive VOCs for each dataset using XAI. We implement the same Machine Learning (ML) workflow, schematized in Fig 2, to each considered dataset separately, as they represent three distinct classification problems involving different sets of VOCs. Since the surrounding environment strongly influences the measurements obtained by sampling breath [19], this approach allows us to evaluate the effectiveness of the same computational analysis procedure in classifying subjects based on VOC abundances collected through various techniques and characterized by different signal-to-noise ratios [20].

To achieve the goal (1), we use leave-one-out cross-validation (LOOCV), a *k*-fold cross-validation with *k* equal to *N*, where *N* is the total number of observations. This validation method is particularly advantageous for small datasets, as it maximizes the size of the training set. The LOOCV process requires *N* iterations, in which N−1 samples are used to train the classifier, and predictions are made on the remaining one. To prevent class imbalance and noise from ML predictions, we apply the SMOTE-ENN algorithm [21] to the training set. This hybrid approach combines oversampling of the minority classes through the Synthetic Minority Over-sampling Technique (SMOTE) with the removal of noisy samples using the Edited Nearest Neighbors (ENN) method. In this study, we adopt a data-driven iterative approach: the SMOTE-ENN procedure is applied once by default, but a second iteration is performed if specific criteria regarding class equilibrium are met. Specifically, this second step is employed when the initial majority class becomes the minority after the first cleaning step, ensuring that any subsequent oversampling of synthetic samples is minimized to prevent the introduction of spurious clusters. This adaptive strategy ensures optimal class balance while preventing the amplification of synthetic noise (see SMOTE-ENN paragraph). We iterate the LOOCV cycle over all samples 100 times, corresponding to the values 1,2,…,100 of the random seed of the ML algorithms. For each iteration, we perform the XAI analysis and record the SHAP values, thus quantifying the impact of VOC abundances on individual predictions to identify potential biomarkers of the considered disorders (goal (2)). Although the scheme in Fig 2 is referred explicitly to the Random Forest (RF), in the present study we explore a wide range of classification algorithms and assess the corresponding performance metrics to identify the optimal one. This analysis is implemented using the PyCaret library (version 3.3.2) [18].

### Performance metrics

Even though accuracy is widely adopted in binary classification and generalizable to the 3-class case, it leads to misleading outcomes when applied to imbalanced datasets [22,23]. Such a situation, commonly called the *accuracy paradox*, occurs when a model achieves a high accuracy value simply by predicting the majority class, but its ability to identify minority classes correctly remains poor. Thus, we focus on the Receiver Operating Characteristic (ROC) curve, which allows us to evaluate the classification algorithms’ effectiveness across different threshold settings. In particular, we calculate the Area Under the ROC Curve (AUC) as a comprehensive measure of classification performance [24].

The mathematical formulation of AUC can be expressed in various ways, depending on the method used for its calculation; the most common one is as follows [25]:


$$\begin{array}{c} AUC=\sum_{i=1}^{n-1}\left(\frac{FPR_{i+1}-FPR_{i}}{2}\times (TPR_{i+1}+TPR_{i})\right), \end{array}$$ (1)

where *n* represents the number of sub-intervals into which the domain of the integral is divided using the trapezoidal method, and *i* is the index that identifies each specific sub-interval or point in the subdivision; *FPR* is the false positive rate, calculated as


$$\begin{array}{c} FPR=\frac{FP}{\left(TN+FP\right)}, \end{array}$$ (2)

with *FP* and *TN* denoting the number of false positives and true negatives, respectively, and *TPR* is the true positive rate


$$\begin{array}{c} TPR=\frac{TP}{\left(TP+FN\right)}, \end{array}$$ (3)

with *TP* and *FN* the numbers of true positives and false negatives, respectively.

The definition of AUC in Eq. (1) has been applied to measure the performance in the binary classification problems. In the 3-class framework, we quantify ML models’ performances using the AUC one-versus-rest (AUC OvR) metrics and the top-2 accuracy. AUC OvR evaluates multi-class classification models [26] by setting up, for each class in the dataset, a binary classification problem in which the model is trained to distinguish between that specific class and all the others. We perform the multi-class AUC calculation for each LOO loop list of predictions, obtaining 100 AUC values at the end of the 100 repetitions, for several ML classifiers. We then compute the mean and standard deviation of AUC values, and identify the best performing classifier for each dataset. Hence, it is possible to construct a ROC curve for each class by calculating the TPR and FPR at various threshold levels. As for the binary AUC, AUC OvR values can range from 0 to 1, where 1 indicates perfect classification, and 0.5 indicates performance equivalent to random guessing.

On the other hand, top-2 accuracy is a performance metric used in the multi-class framework, particularly where models must identify one class among several possible options [27]. While in the binary classification conventional accuracy considers only the most probable prediction, in multi-class problems top-2 accuracy evaluates whether the true label appears among the two most probable predictions returned by the ML classifier. Top-2 accuracy is beneficial in complex classification problems [28] as it provides a broader understanding of models’ performance [29]. Additionally, this approach helps mitigate issues related to class imbalance and overlapping feature values among classes [30].

### SMOTE-ENN

SMOTE-ENN (Synthetic Minority Over-sampling Technique combined with Edited Nearest Neighbors) is a hybrid method, originally proposed by Batista et al. [31], to address class imbalance and reduce noise in datasets [32,33]. It combines the SMOTE and ENN techniques, each serving a distinct purpose in improving the dataset used to train the ML model [34,35]. The first step is SMOTE, an oversampling technique used to generate synthetic samples for the minority class, i.e., the one including fewer samples in a binary classification. SMOTE creates new instances by interpolating between existing ones, increasing the representation of the minority class without merely replicating existing data. Specifically, SMOTE selects a minority class sample and identifies its nearest neighbors within the same class; a new synthetic sample is thus generated by randomly choosing a point along the line segment that connects the selected sample to one of its neighbors. In the case of a multiclass problem, it is possible to set the hyperparameters of the SMOTE algorithm such that all classes but the majority undergo oversampling. The second step is the ENN cleaning technique, which removes noisy and misclassified instances from the dataset based on simulating the nearest neighbor classification, such that misclassified samples are regarded as noisy and marked for removal. ENN helps reduce the overlap between classes, enhancing their separability and refining the decision boundary profile in the feature space. Besides increasing the size of one or more minority classes, the SMOTE-ENN framework reduces noise in the dataset by removing potentially misleading samples. This hybrid approach is applied to balance and clean the training set, which typically results in improved classifier performance.

In this study, we implement an adaptive SMOTE-ENN framework. While the algorithm is often applied once, we evaluate the benefit of a second iteration to further facilitate the removal of noisy samples and achieve better class equilibrium. However, a second application is not universally beneficial and follows a strict decision rule: it is implemented only when the initial majority class becomes a minority after the first ENN cleaning step and, crucially, only if this second pass does not involve a relevant oversampling of previously generated artificial instances. This conditional approach prevents the creation of artificial clusters and spurious patterns that do not represent biological signals. The choice between a single or double iteration is thus determined for each dataset based on these criteria. We adopt the Python Imblearn package (version 0.12.3) [36], setting the k-neighbors hyperparameter to 5 for the creation of synthetic samples.

### Random forest

As we shall see in the Results section, the Random Forest (RF) algorithm compares favorably with the others across the three datasets. Therefore, we implement the ML pipeline shown in Fig 2 using this classifier. The random forest (RF) algorithm is an ensemble learning method that employs multiple decision trees generated through bootstrapping, a procedure in which the training dataset is repeatedly resampled with replacement [37]. The randomization of features during the training phase ensures a low mutual correlation between the trees within the forest. Each decision tree independently predicts the outcome for each observation, and the results are then aggregated, either by averaging in regression tasks or by majority voting for classification. RF is one of the most employed ML algorithms due to several advantages such as ease of tuning, robustness against over-fitting, ability to assess feature importance during training, and unbiased generalization error estimation. In this study, we tune the RF hyperparameters to optimize performance in the 3-class classification tasks on the Lung Cancer, Respiratory Diseases, and IBD datasets, and finally choose the following configuration:

- number of estimators = number of samples in prediction
- criterion = entropy
- max depth = 5.

Setting the number of decision trees equal to the number of samples under examination for each dataset helps mitigate overfitting. The RF develops as a result of several splittings which aim at reducing the entropy of each branching node *m*


$$\begin{array}{c} ENTROPY_{m}=-\sum_{i=1}^{n}p_{mi}log_{2}(p_{mi}), \end{array}$$ (4)

where $p_{mi}$ represents the relative frequency of class *i* observations in node *m*, and *n* is the total number of classes in the dataset. The entropy of a branching node in a RF decision tree quantifies the amount of impurity, namely the heterogeneity of class labels in the subset of instances associated with the node: it equals zero when the subset consists of samples belonging to a unique class and increases with the class variety. The RF growth is controlled by the Information Gain, a parameter that measures the effectiveness of a given split as the related decrease in entropy [38]. The Information Gain drives the tree growth process towards the splits that generate the purest subsets. Finally, the max depth parameter limits the number of iterations of the splitting process: setting its value to 5 provides a good balance between bias and variance, allowing to conjugate the ability to fit the training data and generalize to the test set. The RF algorithm has been implemented using the Python (version 3.9) package Sklearn [39].

### eXplainable Artificial Intelligence (XAI)

The eXplainable Artificial Intelligence (XAI) framework includes various techniques emphasizing informativeness, uncertainty estimation, generalization, and transparency. Among those techniques, we use SHapley Additive exPlanations (SHAP), a model-agnostic posthoc local explanation method based on Shapley values which originates from cooperative game theory [40,41]. We employ the SHAP algorithm to determine the importance of VOCs in both the one-vs-all and binary classifications. The SHAP method implements interpretable linear models for each instance, highlighting the contribution of each feature to its prediction. The SHAP value for a particular (feature, instance) pair is obtained by assessing the difference in the model’s prediction with and without that feature. Such computation involves multiple training of the model on every possible subset *F* of the complete feature set *S*. Specifically, if $f_{x}(F)$ is the model’s prediction for instance *x* using a subset *F* that excludes, for example, the *j*-th feature, and $f_{x}(F\cup \{j\})$ is the prediction when the *j*-th feature is included, the marginal contribution of the *j*-th feature is calculated as the difference $f_{x}(F\cup \{j\})-f_{x}(F)$. The SHAP value for the *j*-th feature in instance *x* is then obtained by summing this difference over all possible subsets:


$$\begin{array}{c} SHAP_{j}(x)=\sum_{F\subseteq S-\{j\}}\frac{|F|!(|S|-|F|-1)!}{|S|!}[f_{x}(F\cup \{j\})-f_{x}(F)], \end{array}$$ (5)

where |*F*|! and (|S|−|F|−1)! represent the number of permutations of features in the subsets *F* and S−(F∪{j}), respectively, and |*S*|! is the total number of feature permutations [42]. In the present study, we apply the XAI analysis to the outcomes provided by the best-performing RF classifier for each of the considered datasets.

SHAP summary plots provide a very effective way to visualize the XAI outcomes. In such representations, each data point corresponds to a specific instance and indicates the SHAP value (horizontal coordinate) for a given feature (row name). SHAP values quantify the impact of each feature on the prediction for a given sample. The interpretation of such plots in the case of binary classification is straightforward: a feature’s contribution towards predicting a class encoded with the 0 or 1 label corresponds to a negative or positive SHAP value, respectively. On the other hand, using the SHAP analysis in the context of a multi-class problem requires the adoption of a one-vs-rest framework, in which the dataset is binarized into two categories: the class of interest and the joint set of the others, called the rest. In summary plots, features are ranked in descending order according to their mean absolute SHAP value across all observations. The color of each point represents the value of the corresponding feature. Therefore, a pronounced chromatic separation in the distribution of SHAP values for a given feature corresponds to a situation of high interpretability, in which the considered feature consistently impacts the classifier’s predictions across all instances. Hence, the color and position of points in the SHAP summary plot are critical elements for interpreting the contributions of different features to the predictions made by ML models. In this study, the SHAP value computation is implemented in the Python (version 3.9) package Shap [43] (version 0.41.0).

## Results

### Performance of the ML classifier

The ML workflow described in the Materials and methods section and shown in Fig 2 has been implemented using different algorithms; classification performances for each dataset are displayed Supplementary S1, S2, and S3 Tables. Although the majority of the considered algorithms achieve comparable performances, RF is the one which, on average, provides slightly better outcomes across the three datasets. In the following, we will refer specifically to the results obtained with RF. Table 2 reports the AUC values RF achieved for each dataset. RF returns an AUC of 0.956 ± 0.013 and 0.914 ± 0.002 for the Respiratory Diseases dataset and the Lung Cancer dataset, respectively. On the other hand, the considered ML algorithms return lower performances for the IBD dataset, reaching an AUC of 0.609 ± 0.013 with RF.

**Table 2:** Performance of RF in 3-class classification, expressed as the mean AUC and its standard deviation, computed over 100 iterations of the computational workflow, corresponding to different random seeds (see Performance metrics paragraph for more detail). The performances of the other ML models are shown in Supplementary S1, S2, S3 Tables.

Dataset	RF performance (AUC)
Lung Cancer	0.914 ± 0.002
Respiratory Diseases	0.956 ± 0.013
Inflammatory Bowel Disease (IBD)	0.609 ± 0.013

**Fig 3:**
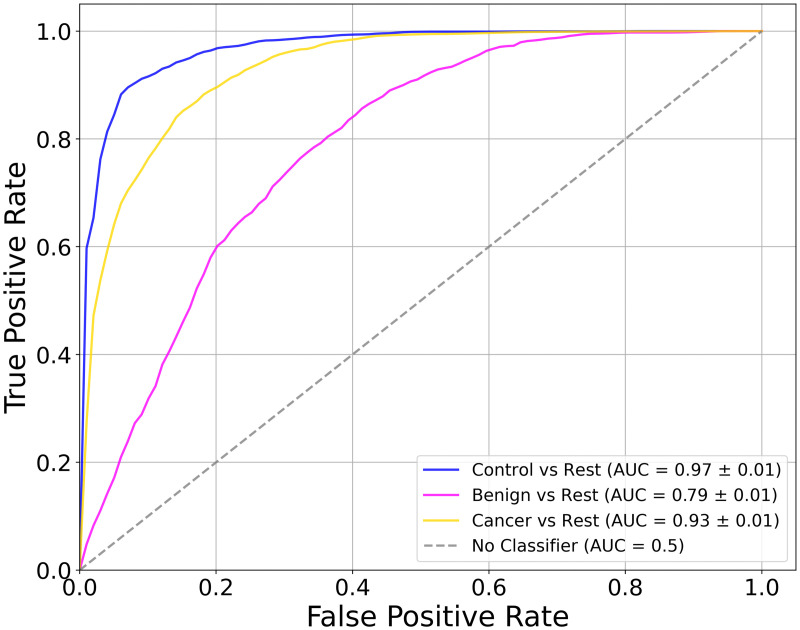
ROC one-vs-rest curves of RF performances in 3-class classification for the Lung Cancer dataset. The legend in panel includes mean AUC values and their standard deviations, computed over 100 iterations of the ML workflow, with the seed being changed at each iteration.

**Fig 4:**
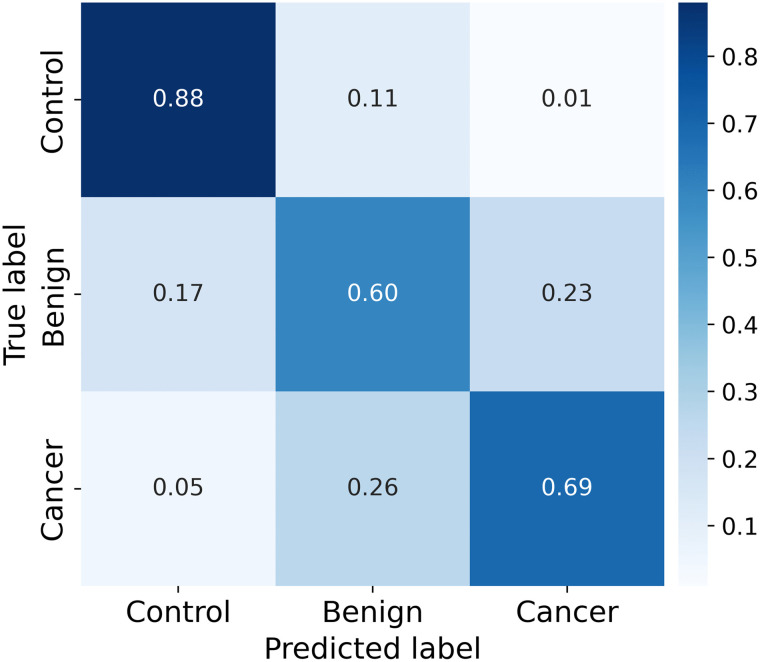
Confusion matrix of RF performances in 3-class classification for the Lung Cancer dataset.

**Fig 5:**
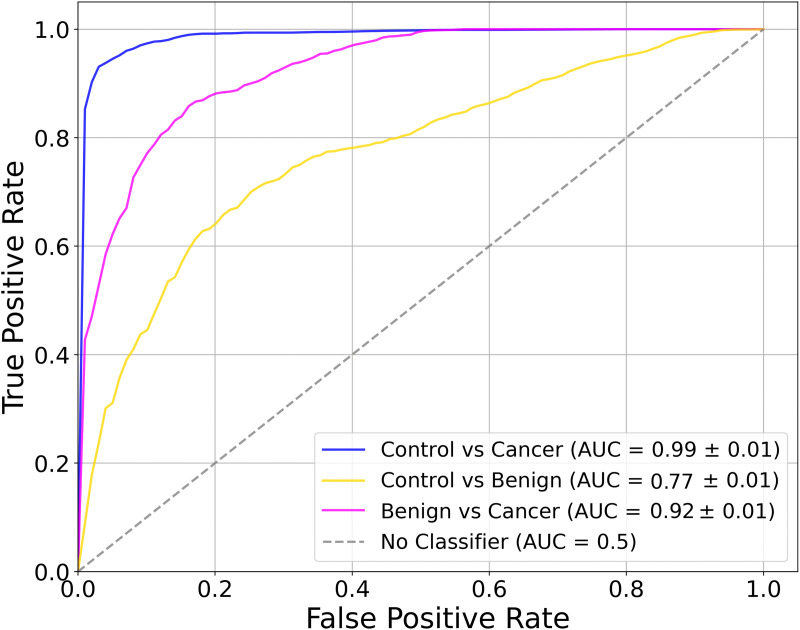
ROC curves of RF performances in binary classifications for the Lung Cancer dataset.

**Fig 6:**
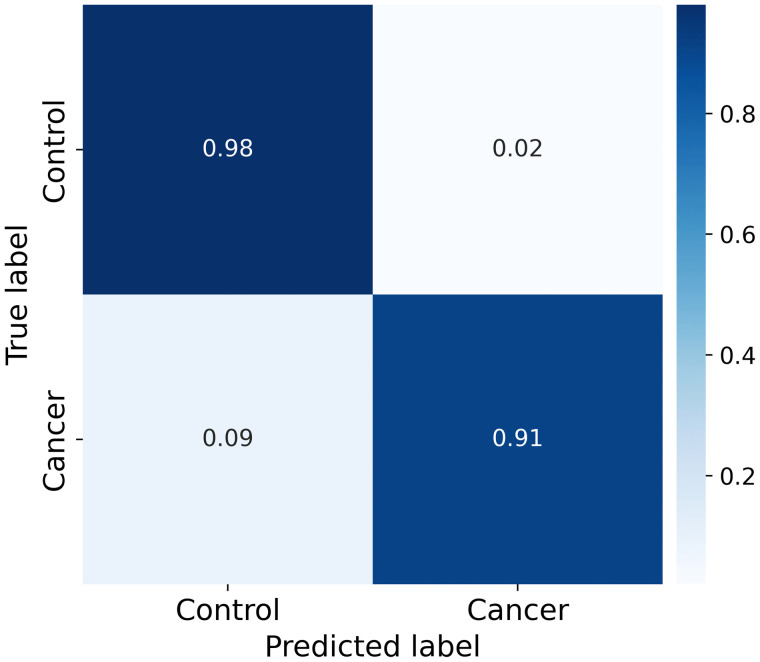
Confusion matrix of RF predictions in the control-vs-cancer classification for the Lung Cancer dataset.

**Fig 7:**
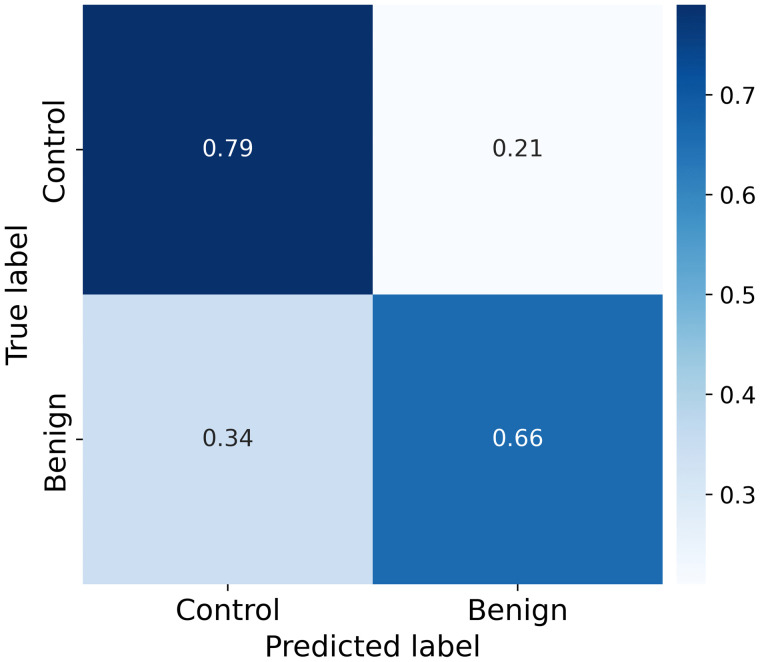
Confusion matrix of RF predictions in the control-vs-benign classification for the Lung Cancer dataset.

**Fig 8:**
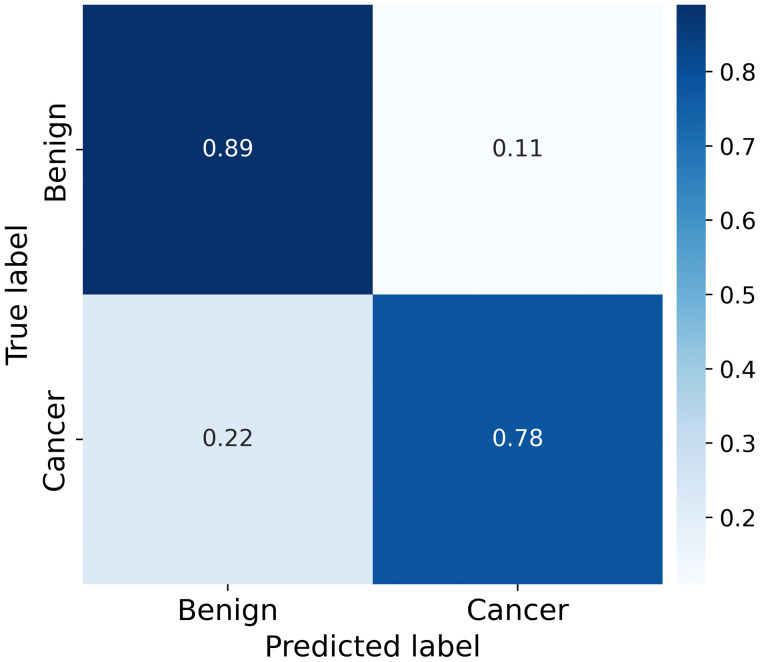
Confusion matrix of RF predictions in the benign-vs-cancer classification for the Lung Cancer dataset.

We report in Fig 3 the ROC curves of RF 3-class classification performance for the Lung Cancer dataset. Each curve is computed by evaluating the predictive rate of one class against the other two combined (one-vs-rest approach), assessing the corresponding AUC in this case. On the other hand, the confusion matrix presented in Fig 4 illustrates the RF’s ability to correctly identify each of the considered classes evaluated independently. To further investigate the discriminative power of the model, we also perform binary classifications between pairs of classes. The ROC curves for the “Control vs Cancer”, “Control vs Benign”, and “Benign vs Cancer” comparisons are shown in Fig 5, which includes the respective AUC values. These binary evaluations provide deeper insight into the specific challenges of distinguishing between different clinical conditions. Finally, the confusion matrices for these binary tasks, presented in Figs 6–8, detail the model’s performance in isolating specific clinical states from healthy controls and from each other.

**Fig 9:**
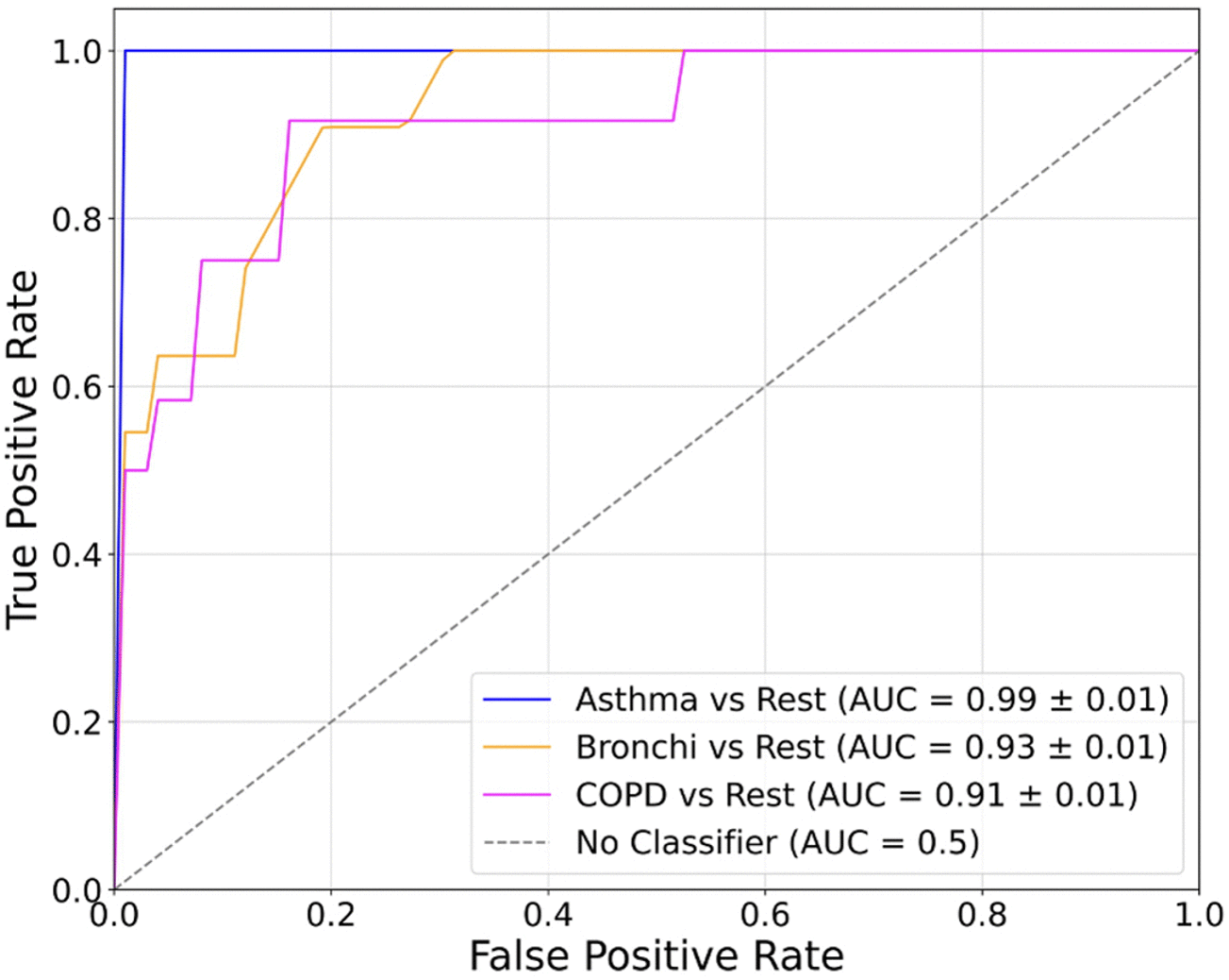
One-vs-rest ROC curves illustrating RF performance in the 3-class classification for the Respiratory Diseases dataset. The legend reports the mean AUC values and standard deviations calculated over 100 iterations of the ML workflow, with a different seed used for each run. For visual clarity, “Bronchiectasis” has been abbreviated as “Bronchi”.

**Fig 10:**
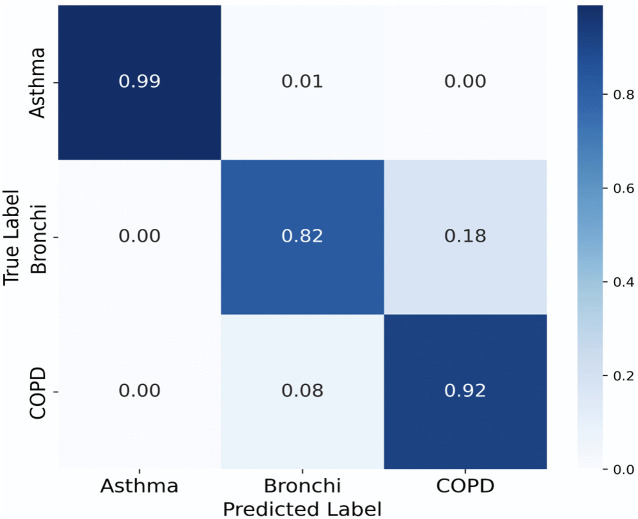
Confusion matrix of RF performances in 3-class classification for the Respiratory Diseases dataset. For visual clarity, “Bronchiectasis” has been abbreviated as “Bronchi”.

**Fig 11:**
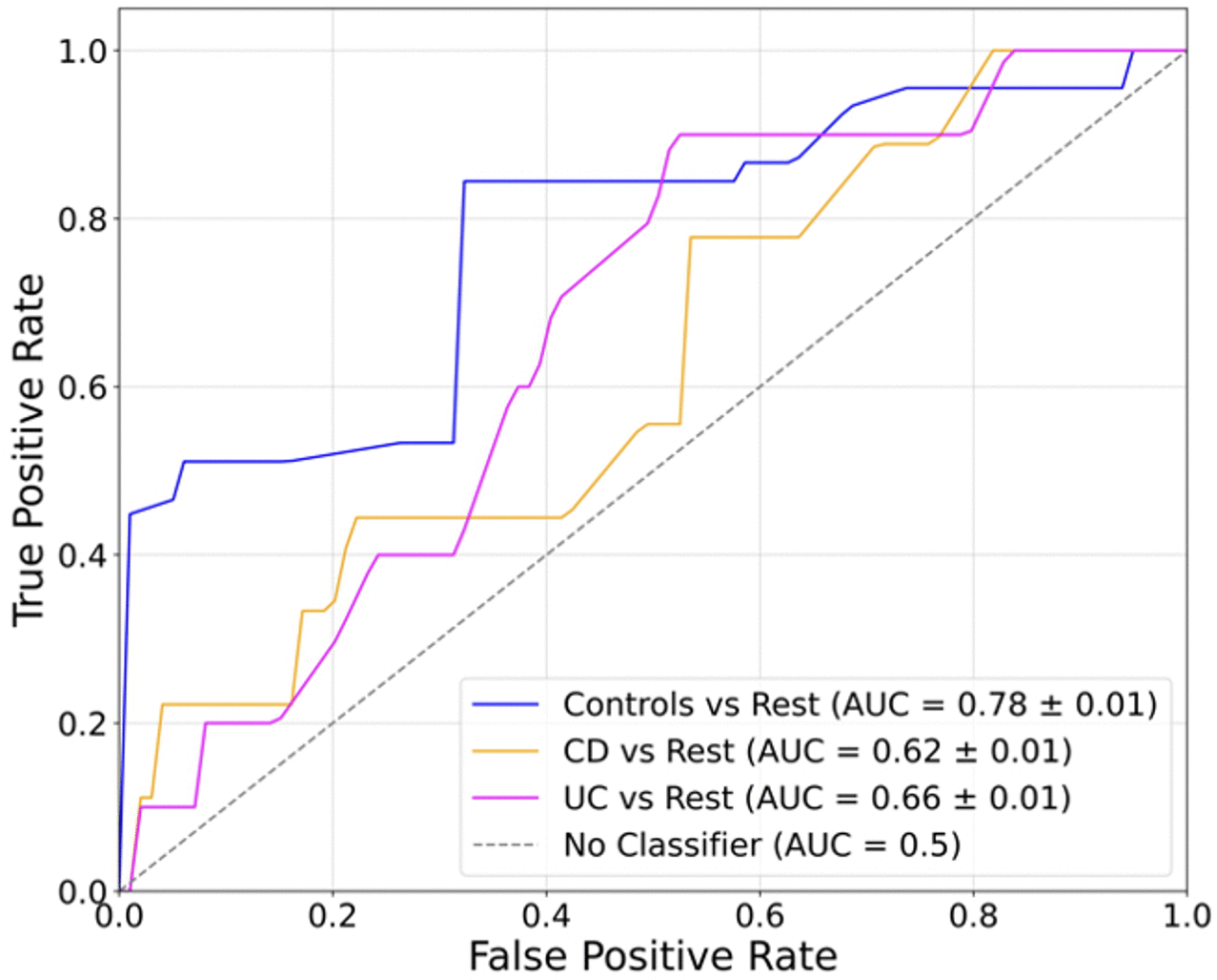
ROC one-vs-rest curves of RF performances in 3-class classification for the IBD dataset. The legend in panel includes mean AUC values and their standard deviations, computed over 100 iterations of the ML workflow, with the seed being changed at each iteration.

**Fig 12:**
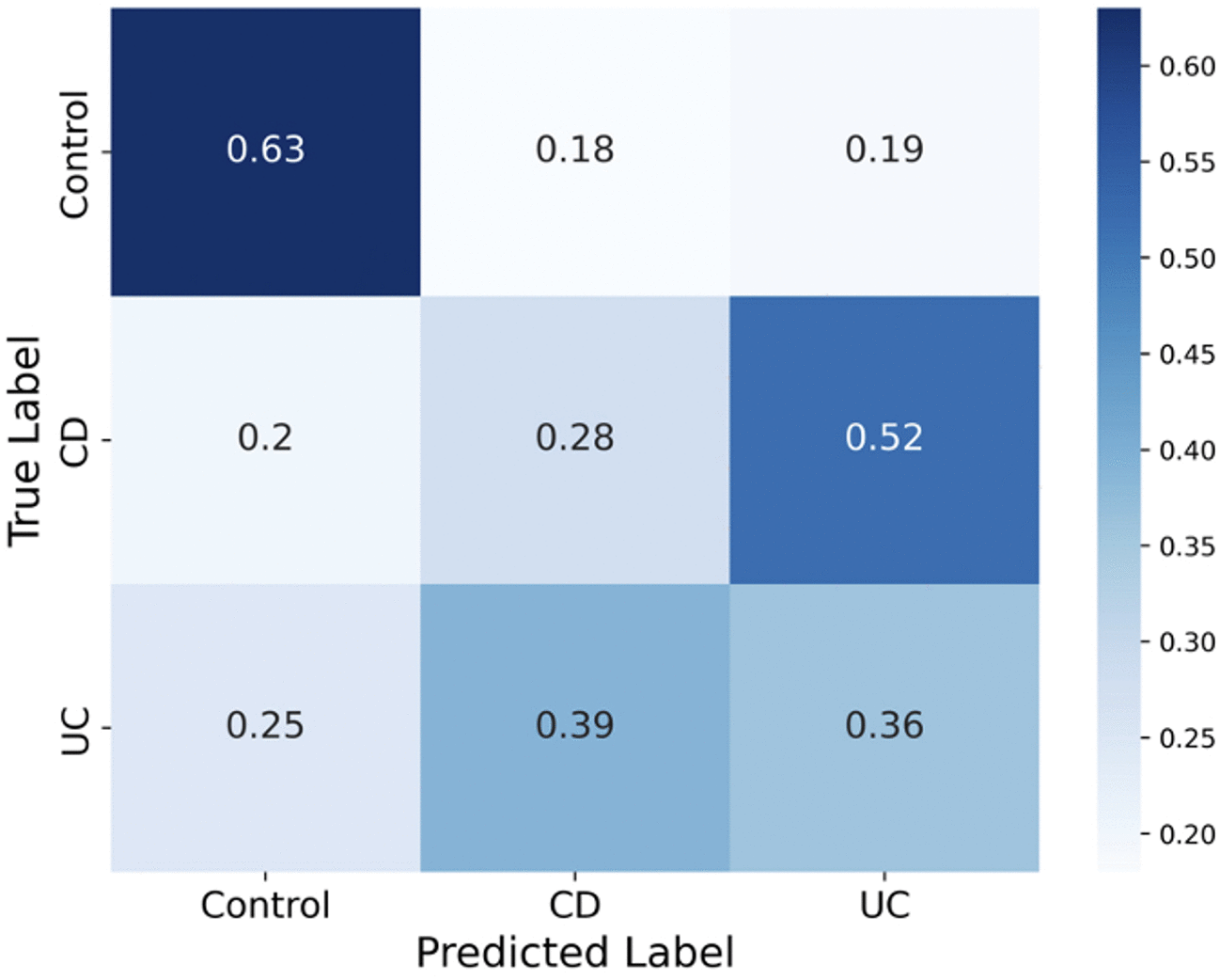
Confusion matrix of RF performances in 3-class classification for the IBD dataset.

Figs 9 and 10 report the ROC curves and confusion matrices for the RF 3-class classification performance of the Respiratory Diseases dataset, evaluated through a one-vs-rest approach. Similarly, the corresponding ROC curves and confusion matrices for the Inflammatory Bowel Disease (IBD) dataset are shown in Figs 11 and 12.

While the classification performances for the Lung Cancer and Respiratory Diseases datasets are generally high, the performance on the IBD is comparatively lower: RF manages to identify 63% of controls while struggles to distinguish between CD and UC labels. As explained in the Discussion, this effect is because CD and UC induce similar metabolic changes, detectable through breath analysis. These changes produce patterns that can effectively distinguish controls from subjects with an inflammatory bowel disease, either CD or UC, considered as a unique group. However, they do not allow for clear differentiation between the two diseases themselves.

### Identification of the most relevant VOCs using XAI

XAI analysis enables the quantification of each VOC’s contribution to the predictions made by the ML algorithm, thereby identifying the most important molecules associated with each disease. As detailed in the eXplainable Artificial Intelligence (XAI) paragraph in the Materials and methods section, the SHAP plot displays features in descending order of importance, from top to bottom. Each point in a SHAP plot corresponds to a specific pair of prediction and feature and is colored according to the feature’s value; the horizontal coordinate of the point reflects the SHAP value, representing the impact of the feature on the prediction. Specifically, positive and negative SHAP values push the classifier towards assigning labels 1 and 0, respectively.

In this study, our focus is not on providing a metabolic or clinical interpretation of the compounds identified as most important. Instead, we aim to offer a methodological tool that can assist clinicians in making and interpreting diagnoses. The SHAP values derived from our analysis help clinicians understand the rationale behind the model’s decisions, enhancing transparency and trustworthiness. Additionally, these values highlight the VOCs that play the most significant role in the predictions, supporting informed decision-making, particularly when determining treatment plans, diagnostic pathways, or personalized treatment approaches.

In the following, we refer to VOCs using their own chemical formula and compound identifier (CID) recorded by the PubChem Library of the American National Library of Medicine [44].

### Lung cancer

**Fig 13:**
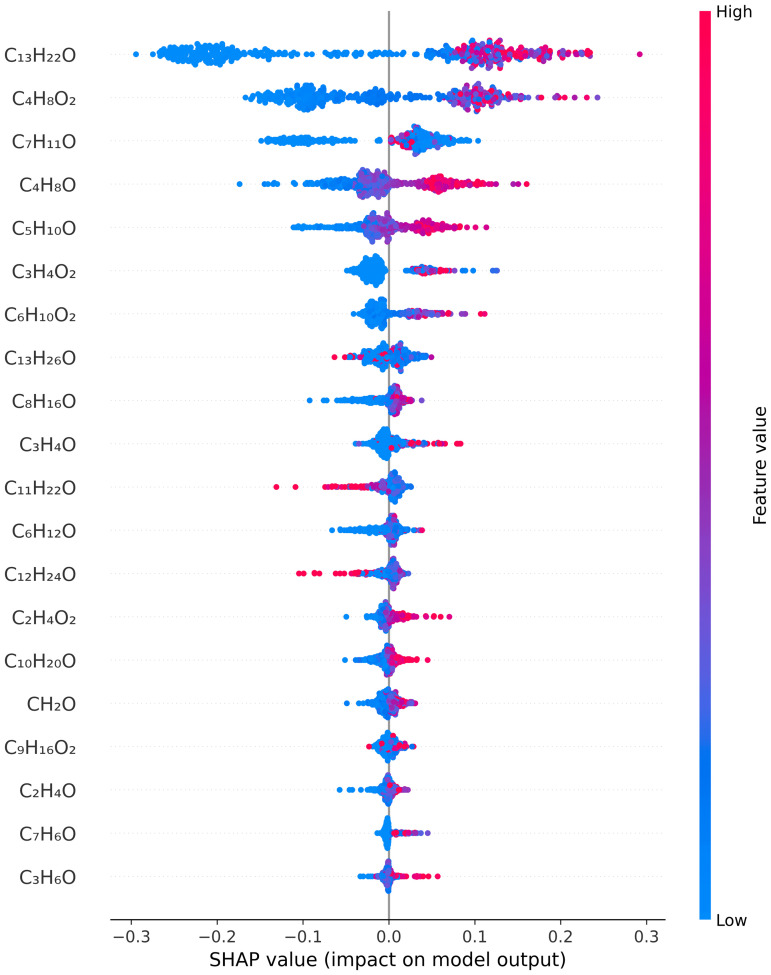
SHAP values for the one-vs-rest classification of the Lung Cancer dataset obtained using RF. This SHAP plot refers to the classification of healthy controls (class 0) vs the set of subjects with benign pulmonary nodules or lung cancer (class 1).

**Fig 14:**
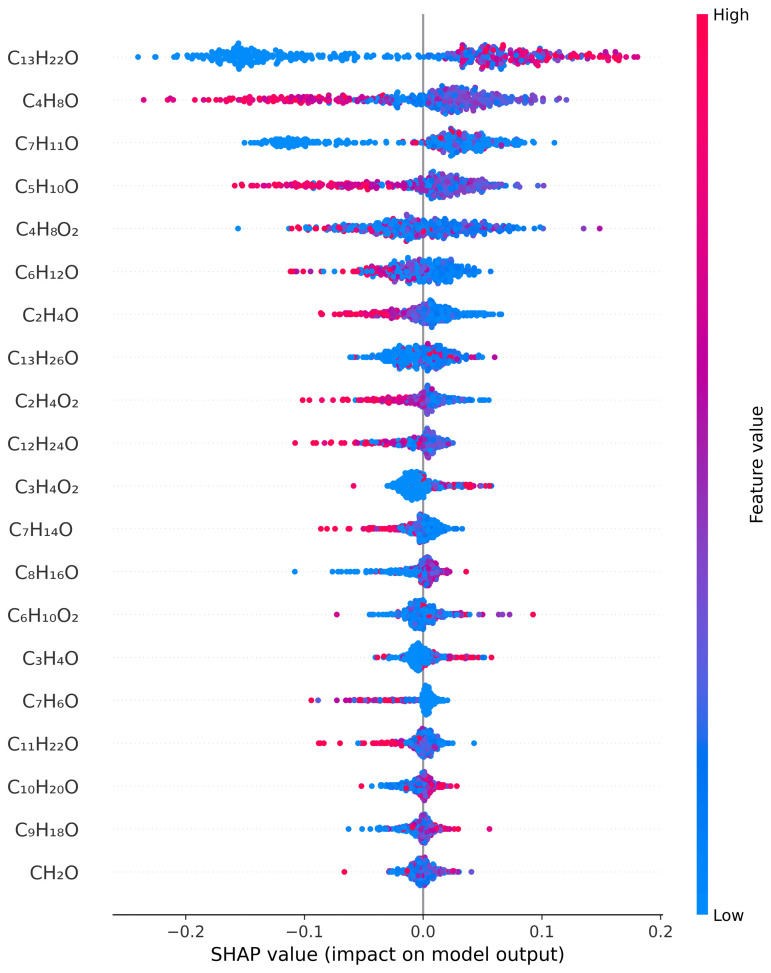
SHAP values for the one-vs-rest classification of the Lung Cancer dataset obtained using RF. This SHAP plot refers to the classification of subjects with benign pulmonary nodules (class 1) vs the set consisting of healthy controls and subjects with lung cancer (class 0).

**Fig 15:**
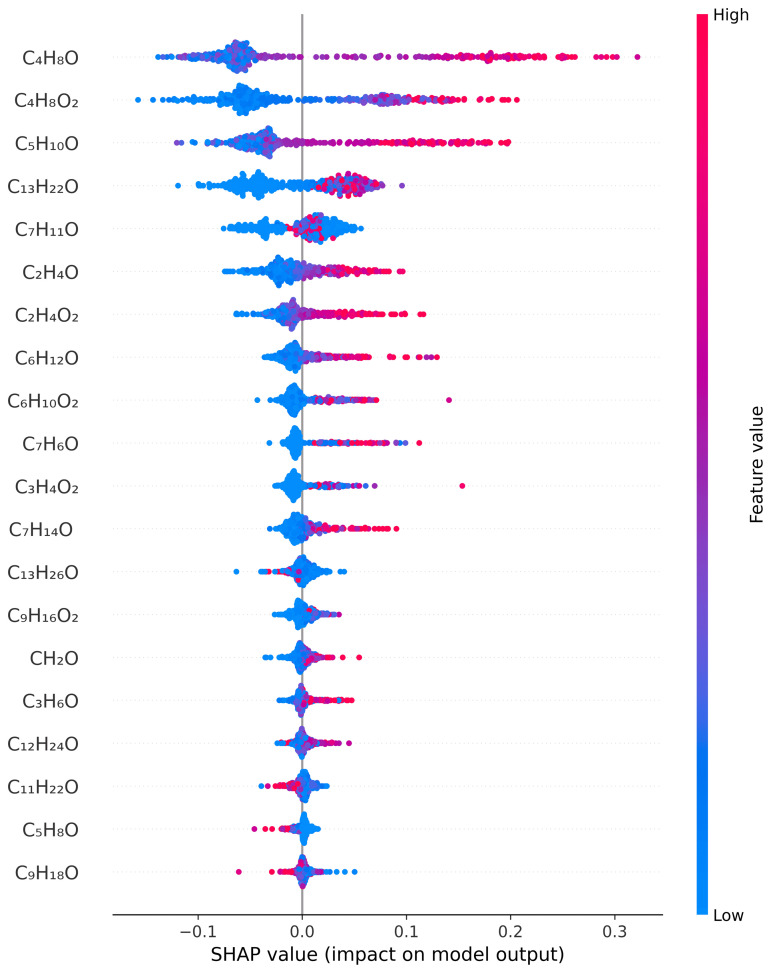
SHAP values for the one-vs-rest classification of the Lung Cancer dataset obtained using RF. This SHAP plot refers to the classification of subjects with lung cancer (class 1) vs the set of healthy controls and subjects with benign pulmonary nodules (class 0).

**Fig 16:**
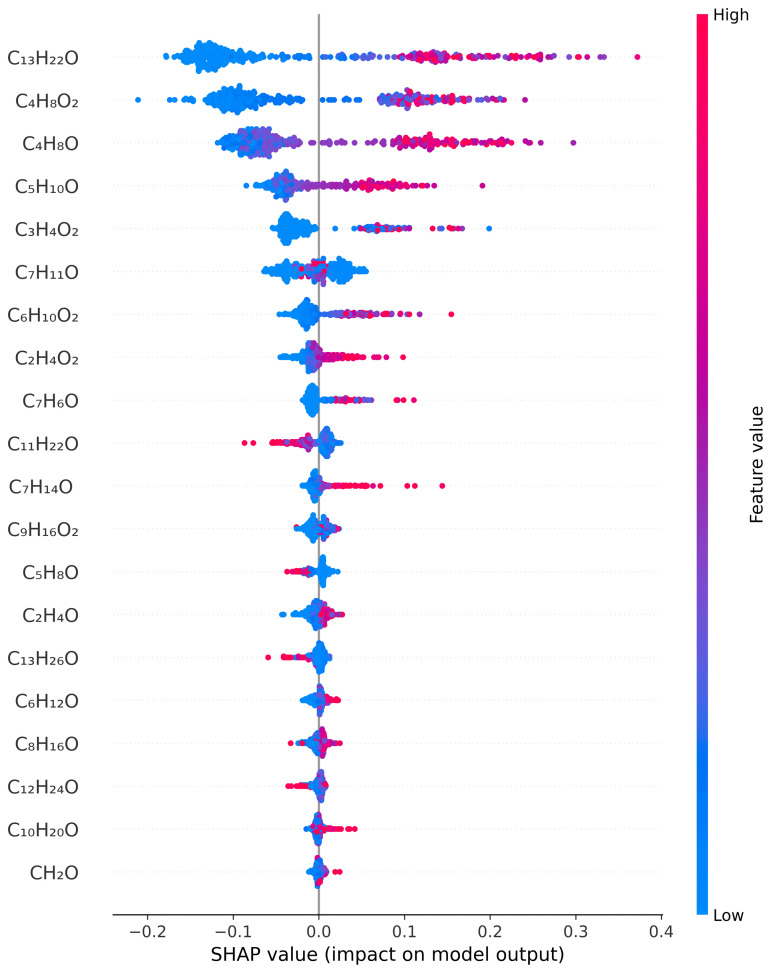
SHAP values for the binary classification of the Lung Cancer dataset obtained using RF. This SHAP plot refers to the classification of healthy controls (class 0) vs the set of subjects with lung cancer (class 1).

**Fig 17:**
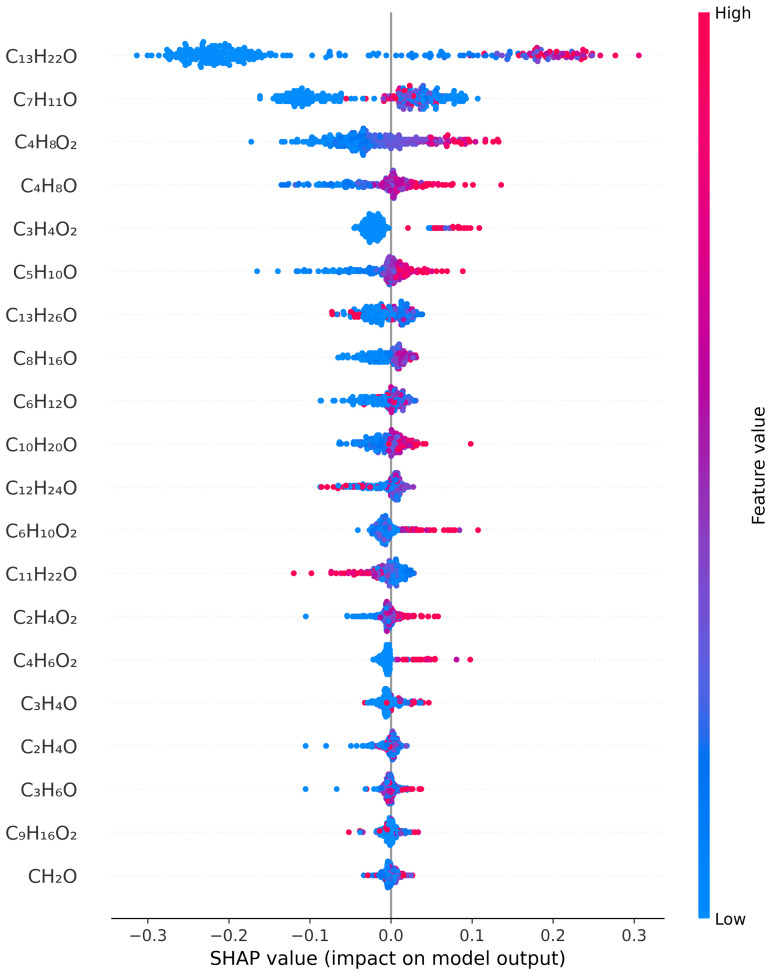
SHAP values for the binary classification of the Lung Cancer dataset obtained using RF. This SHAP plot refers to the classification of healthy controls (class 0) vs the set of subjects with benign pulmonary nodules (class 1).

**Fig 18:**
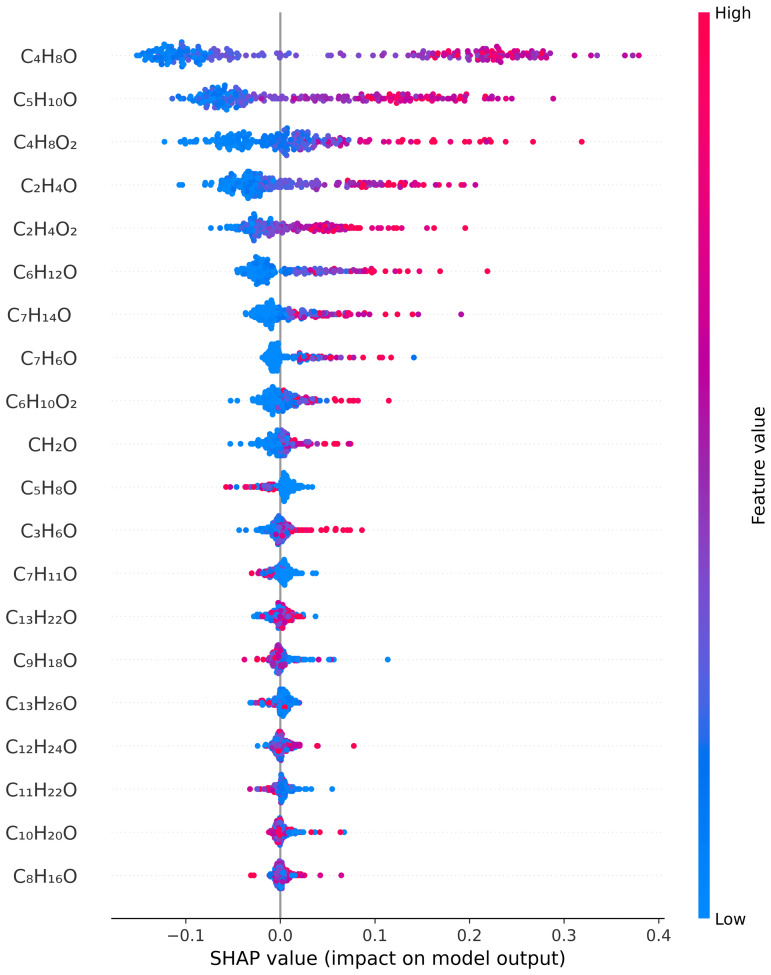
SHAP values for the binary classification of the Lung Cancer dataset obtained using RF. This SHAP plot refers to the classification of subjects with benign pulmonary nodules (class 0) vs the set of subjects with lung cancer (class 1).

The SHAP values of the 20 most important features in the one-vs-rest classification of the Lung Cancer dataset are shown in Figs 13–15, while Figs 16–18 illustrate the top 20 features and their SHAP values for each binary classification within the same dataset [5]. The VOCs with the highest mean absolute SHAP values are compounds belonging to the ketone and aldehyde groups, such as butyraldehyde (C${\;}_{4}{\text{H}}_{8}$O, CID 261), 2-pentanone (C${\;}_{5}{\text{H}}_{10}$O, CID 7895) and hexanal (C${\;}_{6}{\text{H}}_{12}$O, CID 6184).

The important VOCs and their ranking obtained using our methodology are in agreement with those reported by Rai et al. [5], thus validating our approach. To facilitate a direct comparison between our results and those reported by Rai et al. [5], we have included their original findings in the Supplementary Information. Since both studies are based on the same dataset, we evaluated the consistency of the results by comparing the ranking of feature importance. Specifically, Supplementary S4 Table reproduces data from [5], where VOCs identified as being among the top three most significant features in both our analysis and the original study are highlighted in bold. Additionally, Table 2 from Rai et al. is reported as Supplementary S5 Table to provide the statistical distribution of the most relevant VOCs across each class.

In the classification problem comparing controls to the combined group of benign nodules and cancer patients (Fig 13), the top VOCs identified are dicyclohexyl ketone (C${\;}_{13}{\text{H}}_{22}$O, CID 8403) and butanoic acid (C${\;}_{4}{\text{H}}_{8}{\text{O}}_{2}$, CID 264). Notably, these same VOCs also emerge as the most influential in the study by Rai et al. (Case IV, Supplementary S4 Table). The same holds for the classification of cancer patients versus the combined group of benign and control patients (Fig 15), where C${\;}_{4}{\text{H}}_{8}{\text{O}}_{2}$ is also among most important VOCs in the study by Rai et al. (Case V, Supplementary S4 Table).

The three most important features for the control versus cancer binary classification shown in Fig 16 are the same as those highlighted by Rai et al. (Case I, Supplementary S4 Table, albeit appearing in a different order of importance. This consistency across different feature selection methods reinforces the central role of these specific VOCs in cancer discrimination.

### Respiratory diseases

**Fig 19:**
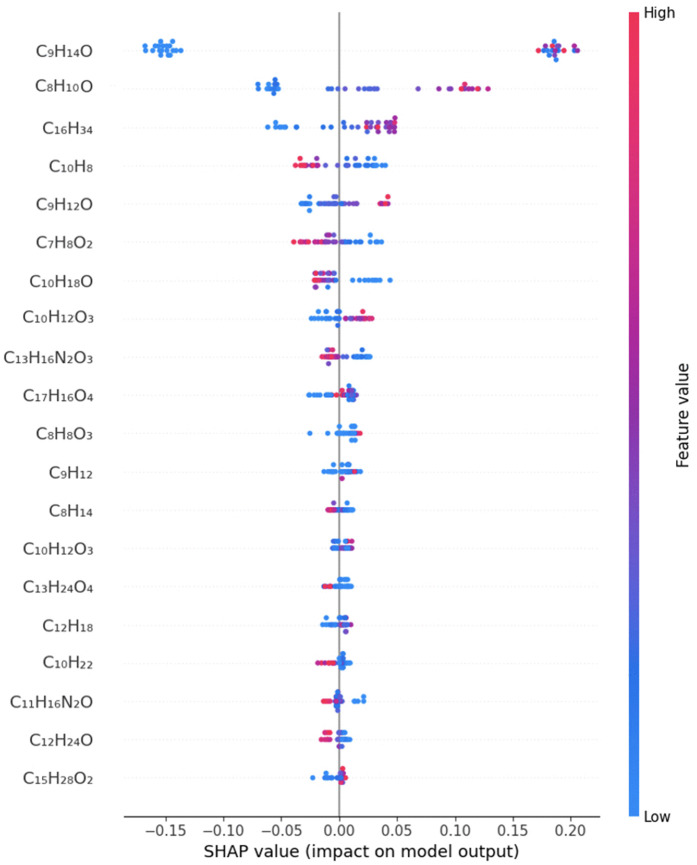
SHAP values for the one-vs-rest classification of the Respiratory Diseases dataset obtained using RF. This SHAP plot refers to the most important VOCs contributing to the classification of asthma patients (label 1) vs the set consisting of COPD and bronchiectasis patients (label 0).

**Fig 20:**
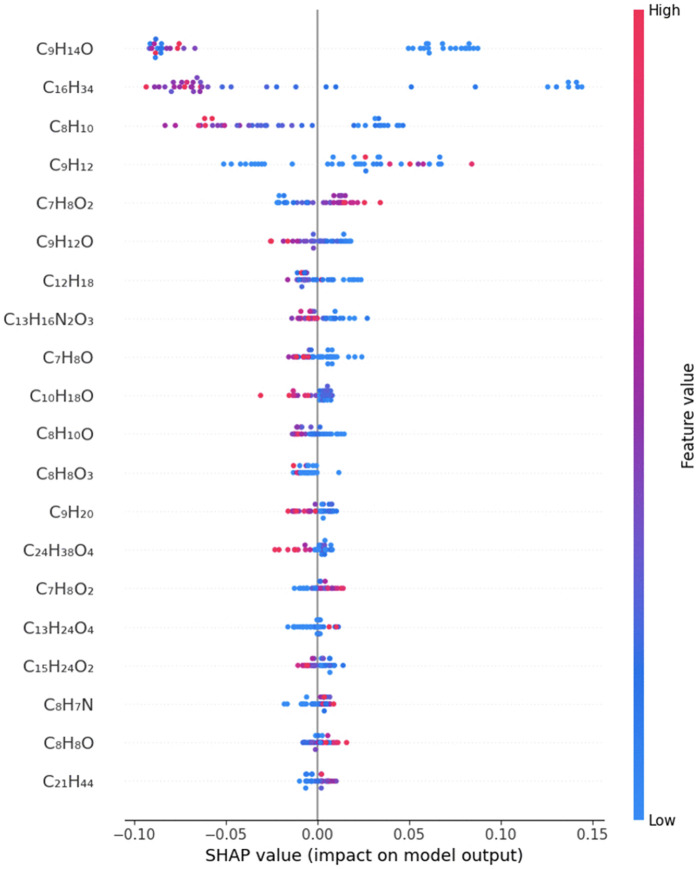
SHAP values for the one-vs-rest classification of the Respiratory Diseases dataset obtained using RF. This SHAP plot refers to the most important VOCs contributing to the classification of bronchiectasis patients (label 1) vs the set consisting of COPD and asthma patients (label 0).

**Fig 21:**
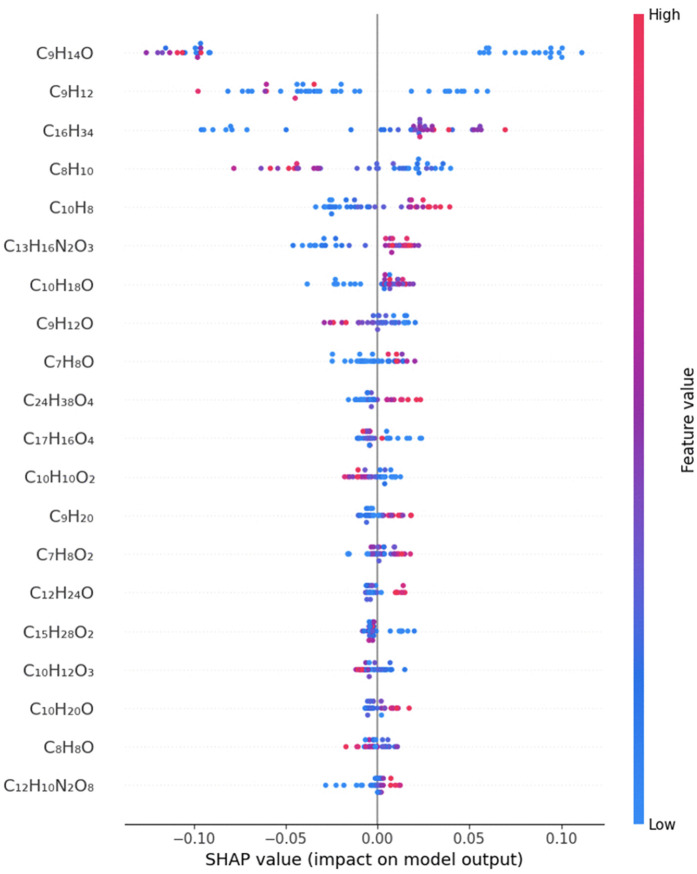
SHAP values for the one-vs-rest classification of the Respiratory Diseases dataset obtained using RF. This SHAP plot refers to the most important VOCs contributing to the classification of COPD patients (label 1) vs the set consisting of bronchiectasis and asthma patients (label 0).

The interpretability of the classification models trained on the Respiratory Diseases dataset [17] is addressed through the SHAP summary plots illustrated in Figs 19–21, which show the impact of individual VOCs on the classification of asthma, bronchiectasis, and COPD. These plots report the SHAP values obtained for each class in a one-vs-rest configuration, representing the contribution of each feature to the model’s output for the three respiratory conditions considered. The most important VOC is 2-pentylfuran (C${\;}_{9}{\text{H}}_{14}$O, CID 19602) belonging to the furan class. High abundances of this compound drive the model to classify the instance as an asthma patient, while lower values lead to classification as COPD or bronchiectasis in the one-vs-rest classification problem. This VOC has been frequently detected in the breath of patients with chronic pulmonary diseases, including asthma and cystic fibrosis, particularly those with Aspergillus fumigatus in their respiratory samples; in contrast, it was not present in the breath of control subjects [45].

Another important VOC in this analysis is hexadecane (C${\;}_{16}{\text{H}}_{34}$, CID 11006). Hexadecane has already been shown to effectively differentiate COPD patients from controls [46]. In our analysis, we find hexadecane ranking third in the SHAP plot shown in Fig 21 for the classification problem COPD vs the jointly group of asthma and bronchiectasis patients, and it ranks second in the SHAP plot displayed in Fig 20, where hexadecane’s lower abundances drive the RF algorithm to recognize patients with bronchiectasis against the other two combined classes (asthma and COPD).

Our literature search did not reveal any clear correlation between the other VOCs identified in our analysis and biomarkers for the respiratory diseases under study. However, this does not rule out the possibility that further research and studies could uncover correlations between these VOCs and specific pulmonary inflammations, such as those observed in our findings.

### Inflammatory bowel disease

**Fig 22:**
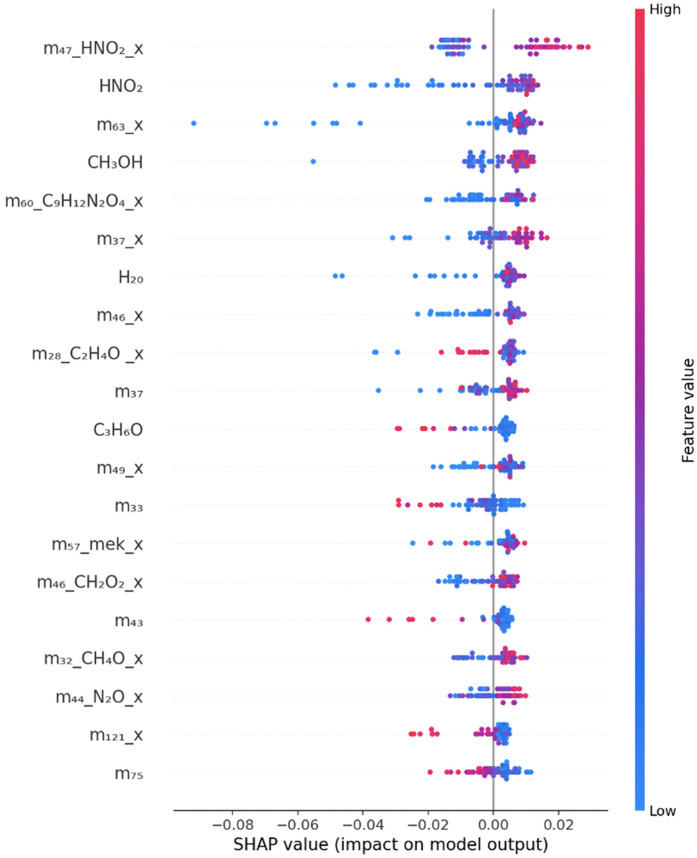
SHAP values for the 3-class classification related to inflammatory bowel disease obtained using RF. This SHAP plot presents the SHAP values in classifying control patients (class 0) vs the set consisting of CD patients and UC patients (class 1).

**Fig 23:**
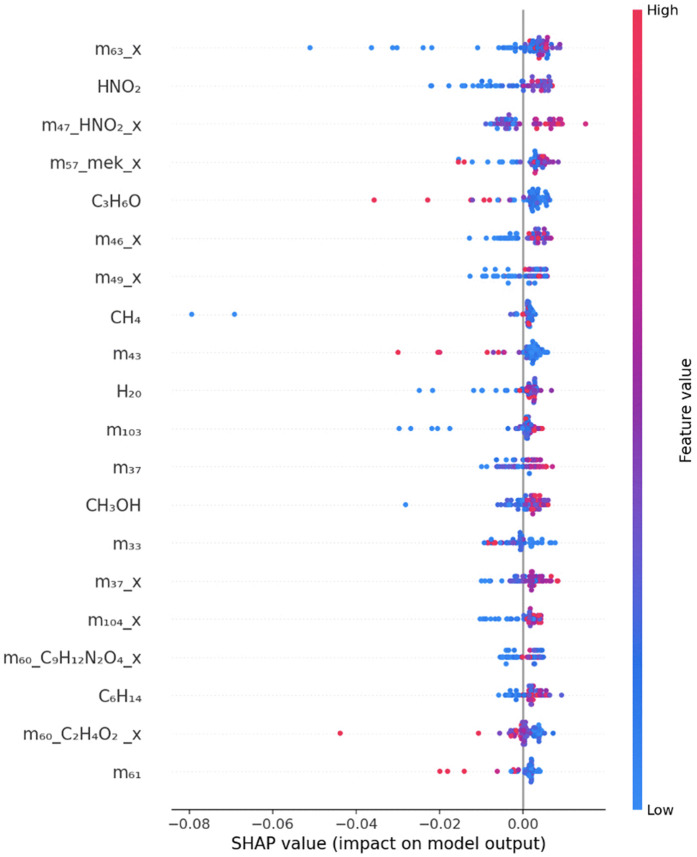
SHAP values for the 3-class classification related to inflammatory bowel disease obtained using RF. This SHAP plot shows the SHAP values in classifying CD patients (class 1) vs the set consisting of controls and UC patients (class 0).

**Fig 24:**
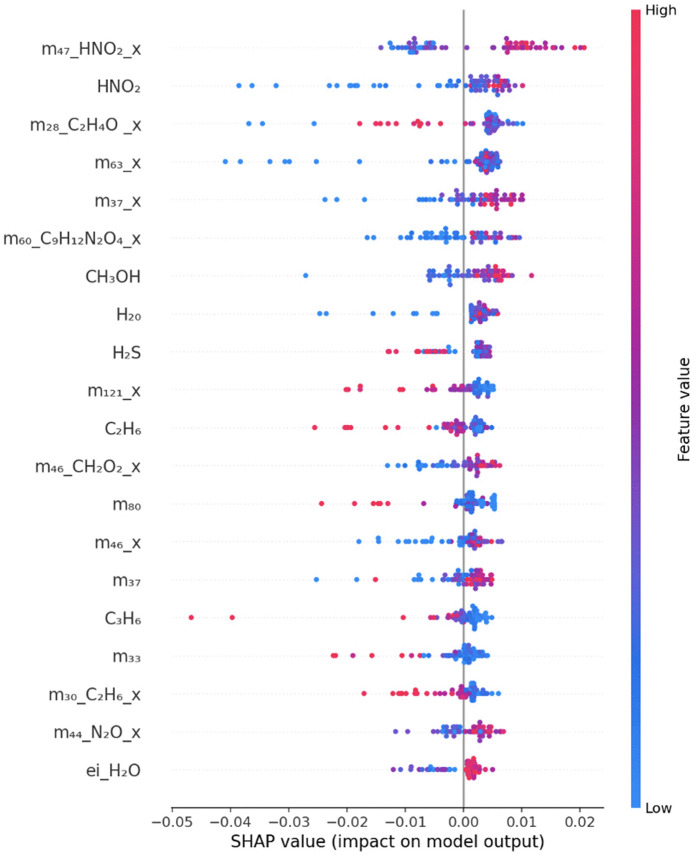
SHAP values for the 3-class classification related to inflammatory bowel disease obtained using RF. This SHAP plot displays SHAP values in classifying UC patients (class 1) vs the set consisting of controls and CD patients (class 0).

The twenty most important features in the one-vs-rest classification of controls, Crohn’s disease, and ulcerative colitis patients in the IBD dataset [7] are shown in Figs 22–24. Notice that, unlike in  Figs 13–21, here VOC names are indicated using a mix of coded chemical names. The reason behind this choice is preserving the notation of the original dataset [7], which includes VOC names referencing molecules with information provided by PubChem [44] or the ChemSpider, i.e., the free online database from the Royal Society of Chemistry [47].

In Figs 22–24, the separation between SHAP values of instances corresponding to high and low abundances appears less pronounced than in Figs 13–15 and Figs 19–21. Moreover, the mean absolute SHAP values of VOCs are typically lower than those found for the Lung Cancer and Respiratory Diseases datasets, reflecting the IBD lower classification performances (see Table 2). Nonetheless, also here some VOCs already known in the literature for their role in altered metabolic processes occupy the top positions in the SHAP rankings.

In particular, we observe the presence of methyl-ketone (indicated in Figs 22–23 as m${\;}_{57}\_\text{mek}\_$x, C${\;}_{3}{\text{H}}_{6}$O, CID 180), which has already demonstrated significance in the classification between controls and a combined group of patients with CD and UC [7]. In particular, an increased abundance has been recorded in subjects with CD or UC, a result also highlighted in the SHAP plot in Fig 22.

Methanol (CH_3_OH, CID 887) emerges as a relevant feature in distinguishing both control subjects (Fig 22) and UC patients (Fig 24) from the other groups. Studies have suggested that the methanol profile may be altered in patients with CD and UC compared to controls, possibly indicating changes in the gut microbiota or intestinal mucosal damage [48].

Regarding the other VOCs returned by the XAI analysis, the existing literature does not seem to associate them with the considered diseases. Nonetheless, further investigations and studies in this field may reveal potential correlations between these VOCs and inflammatory bowel disorders, such as those examined in this work.

## Discussion

Breath analysis holds significant potential due to its non-invasiveness and ease of use. Combined with VOC analysis and AI-based algorithms, it accelerates the screening process and customizes it to the specific pathology under investigation. The strong performance of Random Forest relative to the other machine learning algorithms across each dataset studied is consistent with expectations. The effectiveness of Random Forest stems from its ability to combine the predictions of multiple decision trees, resulting in a robust and accurate model [49]. This algorithm has been widely adopted in various clinical applications, particularly in the development of computer-aided diagnostic systems [50]. Additionally, the design of a pipeline to deal with the challenge of inherently imbalanced datasets, a prevalent obstacle in the application of machine learning algorithms to clinical research and practice, further enhances the value of this study.

The confusion matrices of the Lung Cancer and Respiratory Diseases datasets show that our Random Forest algorithms consistently outperform random chance expectations for 3-class classification tasks. In particular, the proposed model correctly identifies 88% of the controls, distinguishing them from subjects with benign pulmonary nodules or lung cancer (Fig 4). The Random Forest algorithm also demonstrates strong performance in identifying patients with respiratory diseases, accurately detecting nearly all asthma cases and a significant proportion of those with chronic obstructive pulmonary disease (see Fig 10).

The outcomes of the proposed research compare favorably with the literature. In the 2-class discrimination of lung cancer, for instance, our model performs slightly better than other studies on the same subject [51], reaching mean AUC values of 0.99±0.01 (compared to 0.962) and 0.92±0.01 (compared to 0.901) over 100 iterations of the ML workflow in the controls-vs-cancer and benign-vs-cancer classification tasks, respectively.

Regarding respiratory diseases, the literature includes several studies focused on classifying patients affected by one of the three diseases analyzed here (Chronic Obstructive Pulmonary Disease, bronchiectasis, and asthma). One of them classifies COPD, asthma, and normal lung function, capturing VOCs in subjects’ breath through multi-wavelength photo-acoustic sensors [52], returning an AUC of 0.924 for COPD patients classification versus a joint set of asthma and normal lung function patients, and an AUC of 0.928 for asthma patients versus a joint set of COPD and normal lung function patients. This study does not consider bronchiectasis patients. Results achieved in [52] are comparable with those obtained here using the Respiratory Diseases dataset [17] based on the GC–MS VOC acquisition technique, namely AUC values of 0.99±0.01 and 0.91±0.01 in the asthma-vs-rest and COPD-vs-rest predictions, respectively (Fig 9).

Concerning the Inflammatory Bowel Disease dataset, our Random Forest model correctly identifies 63% of controls but fails to distinguish between CD and UC patients effectively (Fig 12). This result highlights a limitation of the proposed model: although CD and UC are two diseases that affect different regions of the intestine (CD can affect any part of the intestinal tract, while UC affects the colon and rectum [53]), the metabolic effects detectable from the VOCs present in [7] appear to be indistinguishable between the two conditions. In fact, the RF classifier struggles to distinguish these two conditions based on the abundances of the VOCs generated by the organism’s biological response to them. On the other hand, our algorithm achieves a better performance in the control-vs-rest classification (AUC = 0.78±0.01 over 100 iterations of the ML workflow, see Fig 11), a task which does not require the identification of patterns discriminating between CD and UC samples. Further insights emerge from the comparison of our results with existing literature. The study proposed by Hicks et al. [54] develops a binary classification framework that returns satisfactory performances not only in the control-vs-CD and control-vs-UC tasks (AUC equals 0.86 and 0.74, respectively) but also in distinguishing between CD and UC patients, with an AUC of 0.83. The performance gap with the outcomes of our analyses can be ascribed to two reasons. Firstly, the IBD dataset examined in the present study consists of VOC samples that have been analyzed using ion molecule mass spectrometry [7], while in Ref. [54], a different breath analysis technique, known as ion flow tube mass spectrometry, is adopted. Secondly, the two studies employ sets of VOCs with a minimal overlap: only 3 out of 26 VOCs analyzed in [54] are also present in the IBD dataset here considered. Such a difference between the outcomes of the two studies demonstrates how sensitive breath analysis is to the specific detection technique and the set of VOCs examined, as not all of them are effective biomarkers to distinguish between the disorders of interest.

Another goal of our research is to enhance the interpretability of ML classifiers by identifying the VOCs whose abundances most significantly influence the predictions. The XAI analysis performed in the present study yields several findings that are supported by existing literature, highlighting the key VOCs generated in the body’s response to disease for each of the three case studies. As shown in Figs 13–15, for instance, the VOCs dicyclohexyl ketone, butanoic acid, and butyraldehyde emerge as important in the lung cancer dataset. These VOCs were also found to play a key role in the study by Rai et al [5]. As regards the analysis of the respiratory diseases dataset, 2-pentylfuran and hexadecane are identified as important VOCs (Figs 19–21). Furthermore, methyl-ketone and methanol impact the classification of inflammatory bowel diseases, as shown in the SHAP plots of Figs 22–24. This study also offers new insights into previously unreported VOCs identified as important by the XAI methodology that remain largely unexplored in the literature and may represent novel potential biomarkers.

The performance of the proposed machine learning workflow, combined with the interpretability of its results, supports the identification of new potential biomarkers for various disorders. However, datasets composed of VOCs collected from breath using different acquisition techniques—such as those examined in this study—can yield markedly different outcomes. This variability is mainly due to the influence of the data collection process on factors such as the signal-to-noise ratio, which can significantly affect model performance and reliability. This heterogeneity highlights the need for standardization in the collection of breath samples, as well as in the detection and quantification of VOCs. Furthermore, selecting an appropriate set of VOCs to input into the ML classifier should be tailored to the disorder of interest in order to enhance the identification of distinctive biomarkers.

Although breath testing offers advantages such as non-invasiveness and simplicity [55], the standardization of sampling, filtration, detection, and quantification of VOCs is essential before proceeding to a clinical implementation [56]. Since the molecules present in breath are highly dependent on the individual’s interaction with the environment [57], further standardization and validation are critical before the widespread clinical adoption of breath analysis [58,59].

## Supporting information

S1 File Supporting information. (PDF)

S2 File List of VOCs for each dataset. To facilitate comparison across datasets, the same color was used to identify the same VOC whenever it appears in different datasets (i.e., across different columns).
(XLSX)

S1 Table Performance of PyCaret classification algorithms for the 3 classes of the Lung cancer dataset. Results of the RF model are highlighted in boldface. (PDF)

S2 Table Performance of Pycaret classification algorithms for the 3 classes of the Clinical Breathomics dataset. Results of the RF model, ranking 4th in AUC and 1st (together with 3 other algorithms) in Top-2 accuracy, are highlighted in boldface.
(PDF)

S3 Table Performance of Pycaret classification algorithms for the 3 classes of the IBD dataset. Results of the RF model, ranking 3rd in AUC and 6th in Top-2 accuracy, are highlighted in boldface.
(PDF)

S4 Table Ranking of significant VOCs across clinical classifications. This table reproduces the ranking of the most important VOCs for binary classification as reported by Rai et al. (2022). Features are ranied based on their computed false discovery rate values. VOCs highlighted in bold represent the top three most significant features consistently identified in both our current analysis and the previous study. The classification scenarios include: Case I (Cancer vs. Control), Case II (Cancer vs. Benign), Case III (Benign vs. Control), Case IV (Cancer + Benign vs. Control), and Case V (Control + Benigh vs. Cancer).
(PDF)

S5 Table Summary statistics of the most relevant VOCs for the three clinical classes (Control, Benign, Cancer) in the Lung Cancer dataset. Data adapted from Rai et al (2022) to provide a reference for the feature profiles used in predictive modeling.
(PDF)
