# Supplementary material for: Enhancing breath-based diagnostics through eXplainable Artificial Intelligence
Source: PLoS One. 2026 Jun 26;21(6):e0351833. doi: 10.1371/journal.pone.0351833 (PMC13308859; doi:10.1371/journal.pone.0351833)
Supplement: S1 File — (PDF) [file pone.0351833.s001.pdf]

Additional Data Information

The first dataset, termed *Lung Cancer*, comprises 28 VOCs for 427 subjects, namely 193 healthy controls, 65 patients with benign pulmonary nodules, 153 patients with lung cancer. The second dataset, referred to as *Respiratory diseases*, includes 70 VOCs for 121 subjects: 33 with Chronic Obstructive Pulmonary Disease, 35 with bronchiectasis, and 53 with asthma. The last dataset, focused on *Inflammatory Bowel Disease*, consists of 220 children, 167 of whom are healthy controls, 25 with Crohn's disease (CD), and 28 with Ulcerative Colitis (UC), with 200 VOCs analyzed. Further information on these datasets is reported in the *Material and methods* section in the main text.
We assessed the overlap of VOCs across the three datasets and found that none were common to all three. Partial overlap exists, with some VOCs occurring in two of the datasets. In particular, they are:

- **Formaldehyde**, included in both the Lung Cancer (CH_2O) and IBD (`m30_formaldeide`) datasets
- **Acetaldehyde**, included in both the Lung Cancer (C_2H_4O) and IBD (`m44_acetaldeide`) datasets
- **Acetone**, included in both the Lung Cancer (C_3H_6O) and IBD (`m58_acetone`) datasets
- **Butanone**, included in both the Lung Cancer (C_4H_8O) and IBD (`m57_mek`, i.e. methyl ethyl ketone = butanone) datasets
- **Acetic Acid**, included in both the Lung Cancer ($C_2H_4O_2$) and IBD (`m60_ac_acetico`) datasets
- **Benzaldehyde**, included in both the Lung Cancer (C_7H_6O) and Respiratory Disease datasets
- **Dodecanal**, included in both the Lung Cancer ($C_{12}H_{24}O$) and Respiratory Disease datasets.

The list of VOCs for each dataset has been included in S2_file.xlsx. In this file, each column corresponds to a dataset. To facilitate comparison across datasets, the same color was used to identify the same VOC whenever it appears in different datasets (i.e., across different columns).

Summary of the Machine Learning Workflow

In this study, we apply a Machine Learning (ML) pipeline to develop a classifier that predicts disorders from VOC abundances collected in breath analysis. The proposed workflow, schematized in Fig. 2 of the main text, is independently applied to three datasets. Further information on data engineering is reported in the *Material and methods* section in the main text, where we also describe the procedure to identify the optimal classifier from a list of popular algorithms [1].
The pipeline is based on a leave-one-out cross-validation (LOOCV) framework. To handle class imbalance within the training set, we adopt a data-driven iterative approach based on the SMOTE-ENN procedure. By default, the resampling is applied

in a single stage; however, a second iteration is performed if specific criteria regarding class equilibrium are met. Specifically, this second step is triggered when the initial majority class becomes the minority following the first cleanning phase, and any subsequent oversampling of synthetic samples is minimized. This methodology prevents the introduction of spurious clusters and the amplification of synthetic noise.
We integrate into the workflow several ML classifiers, available in the PyCaret library [1], and identify the ones returning the best performance. This approach ensures that we leverage the strengths of various classification algorithms while maintaining a systematic methodology throughout the modeling process. For each classifier, we record predictions and evaluate the AUC and Top-2 accuracy achieved in the leave-one-out cycle. This procedure is repeated 100 times, assigning values $1, 2, \dots, 100$ to the random seed parameter of the algorithms implemented in the ML workflow. The proposed approach provides us information on the stability of AUC and Top-2 accuracy performance metrics, whose mean values and standard deviation over the 100 iterations are reported in Tables S1 S2 and S3.

Focus on VOCs in the Lung Cancer dataset

To provide a comprehensive context for our predictive results, we include the original findings from Rai et al. [2], who first analyzed the Lung Cancer dataset employed in this study. The inclusion of these outcomes allows for a direct comparison between the VOCs identified by our pipeline and the significant features reported in the literature. Supplementary Table S4 reproduces the ranking of significant VOCs as originally presented in Rai et al. (Table 3 in [2]). In this table, VOCs that were identified among the top three most significant features in both our analysis and the original study are highlighted in bold to indicate consistency across different methodologies. The ranking is organized according to five distinct classification scenarios, defined as follows:

- **Case I:** Cancer (156) vs. Control (193);
- **Case II:** Cancer (156) vs. Benign (65);
- **Case III:** Benign (65) vs. Control (193);
- **Case IV:** (Benign + Cancer) (221) vs. Control (193);
- **Case V:** (Control + Benign) (258) vs. Cancer (156).

Supplementary Table S5 (originally Table 2 in [2]) complements this ranking by providing the summary statistics for the most relevant VOCs. This table details the statistical distribution of these compounds across the three patient populations (Control, Benign Pulmonary Nodule, and Lung Cancer), offering a reference for the baseline abundance levels that characterize each group.

References

[1] Ulla Gain and Virpi Hotti. "Low-code AutoML-augmented data pipeline–a review and experiments". In: *Journal of Physics: Conference Series*. Vol. 1828. 1. IOP Publishing. 2021, p. 012015.

[2] Shesh N Rai et al. "Multigroup prediction in lung cancer patients and comparative controls using signature of volatile organic compounds in breath samples". In: *Plos one* 17.11 (2022), e0277431.
