# Supplementary material for: Enhancing breath-based diagnostics through eXplainable Artificial Intelligence
Source: PLoS One. 2026 Jun 26;21(6):e0351833. doi: 10.1371/journal.pone.0351833 (PMC13308859; doi:10.1371/journal.pone.0351833)
Supplement: S1 Table — (PDF) [file pone.0351833.s003.pdf]

| Model | AUC Performance | Top-2 Acc Performance |
|---|---|---|
| Ada Boost Classifier | 0.858 ± 0.004 | 0.933 ± 0.008 |
| Decision Tree Classifier | 0.762 ± 0.011 | 0.887 ± 0.011 |
| Dummy Classifier | 0.587 ± 0.003 | 0.817 ± 0.005 |
| Extra Trees Classifier | 0.918 ± 0.004 | 0.944 ± 0.009 |
| Extreme Gradient Boosting | 0.903 ± 0.004 | 0.931 ± 0.009 |
| Gradient Boosting Classifier | 0.897 ± 0.006 | 0.930 ± 0.007 |
| K Neighbors Classifier | 0.814 ± 0.007 | 0.893 ± 0.012 |
| Linear Discriminant Analysis | 0.845 ± 0.009 | 0.931 ± 0.009 |
| Logistic Regression | 0.883 ± 0.004 | 0.914 ± 0.005 |
| Naive Bayes | 0.740 ± 0.012 | 0.908 ± 0.009 |
| Quadratic Discriminant Analysis | 0.710 ± 0.010 | 0.869 ± 0.011 |
| **Random Forest Classifier** | **0.914 ± 0.002** | **0.949 ± 0.005** |
| Ridge Classifier | 0.858 ± 0.004 | 0.901 ± 0.005 |
| SVM - Linear Kernel | 0.881 ± 0.006 | 0.939 ± 0.006 |

**Table S1.** Performance of PyCaret classification algorithms for the 3 classes of the Lung cancer dataset. Results of the RF model are highlighted in boldface.
