# Supplementary material for: Enhancing breath-based diagnostics through eXplainable Artificial Intelligence
Source: PLoS One. 2026 Jun 26;21(6):e0351833. doi: 10.1371/journal.pone.0351833 (PMC13308859; doi:10.1371/journal.pone.0351833)
Supplement: S2 Table — Results of the RF model, ranking 4th in AUC and 1st (together with 3 other algorithms) in Top-2 accuracy, are highlighted in boldface. (PDF) [file pone.0351833.s004.pdf]

Model	AUC Performance	Top-2 Acc Performance
Ada Boost Classifier	0.979 ± 0.009	0.990 ± 0.010
Decision Tree Classifier	0.954 ± 0.018	0.989 ± 0.019
Dummy Classifier	0.583 ± 0.041	0.582 ± 0.025
eXtreme Gradient Boosting	0.975 ± 0.011	0.990 ± 0.010
Extra Trees Classifier	0.909 ± 0.021	0.955 ± 0.015
Gradient Boosting Classifier	0.959 ± 0.016	0.990 ± 0.010
K Neighbors Classifier	0.746 ± 0.031	0.837 ± 0.028
Linear Discriminant Analysis	0.880 ± 0.026	0.773 ± 0.046
Logistic Regression	0.913 ± 0.022	0.919 ± 0.012
Naive Bayes	0.837 ± 0.031	0.928 ± 0.012
Quadratic Discriminant Analysis	0.504 ± 0.035	0.700 ± 0.014
**Random Forest Classifier**	**0.956 ± 0.013**	**0.990 ± 0.010**
Ridge Classifier	0.917 ± 0.022	0.87 ± 0.02
SVM - Linear Kernel	0.923 ± 0.020	0.91 ± 0.01

Table S2. Performance of Pycaret classification algorithms for the 3 classes of the Clinical Breathomics dataset. Results of the RF model, ranking 4th in AUC and 1st (together with 3 other algorithms) in Top-2 accuracy, are highlighted in boldface.
