# Supplementary material for: Enhancing breath-based diagnostics through eXplainable Artificial Intelligence
Source: PLoS One. 2026 Jun 26;21(6):e0351833. doi: 10.1371/journal.pone.0351833 (PMC13308859; doi:10.1371/journal.pone.0351833)
Supplement: S3 Table — Results of the RF model, ranking 3rd in AUC and 6th in Top-2 accuracy, are highlighted in boldface. (PDF) [file pone.0351833.s005.pdf]

Model	AUC Performance	Top-2 Acc Performance
Ada Boost Classifier	0.602 ± 0.026	0.656 ± 0.026
Decision Tree Classifier	0.559 ± 0.016	0.580 ± 0.023
Dummy Classifier	0.489 ± 0.018	0.249 ± 0.000
eXtreme Gradient Boosting	0.635 ± 0.010	0.760 ± 0.001
Extra Trees Classifier	0.580 ± 0.015	0.660 ± 0.009
Gradient Boosting Classifier	0.612 ± 0.007	0.717 ± 0.017
K Neighbors Classifier	0.494 ± 0.013	0.504 ± 0.015
Linear Discriminant Analysis	0.572 ± 0.017	0.636 ± 0.025
Logistic Regression	0.550 ± 0.010	0.680 ± 0.010
Naive Bayes	0.565 ± 0.013	0.742 ± 0.014
Quadratic Discriminant Analysis	0.500 ± 0.000	0.883 ± 0.000
**Random Forest Classifier**	**0.609 ± 0.013**	**0.705 ± 0.015**
Ridge Classifier	0.516 ± 0.013	0.532 ± 0.006
SVM - Linear Kernel	0.567 ± 0.012	0.789 ± 0.011

Table S3. Performance of Pycaret classification algorithms for the 3 classes of the IBD dataset. Results of the RF model, ranking 3rd in AUC and 6th in Top-2 accuracy, are highlighted in boldface.
