# Supplementary material for: Enhancing breath-based diagnostics through eXplainable Artificial Intelligence
Source: PLoS One. 2026 Jun 26;21(6):e0351833. doi: 10.1371/journal.pone.0351833 (PMC13308859; doi:10.1371/journal.pone.0351833)
Supplement: S4 Table — This table reproduces the ranking of the most important VOCs for binary classification as reported by Rai et al. (2022). Features are ranied based on their computed false discovery rate values. VOCs highlighted in bold represent the top three most significant features consistently identified in both our current analysis and the previous study. The classification scenarios include: Case I (Cancer vs. Control), Case II (Cancer vs. Benign), Case III (Benign vs. Control), Case IV (Cancer + Benign vs. Control), and Case V (Control + Benigh vs. Cancer). (PDF) [file pone.0351833.s006.pdf]

Rank	Case I	Case II	Case III	Case IV	Case V
1	$\mathbf{C_4H_8O}$	C_5H_8O	$\mathbf{C_4H_8O_2}$	$\mathbf{C_4H_8O_2}$	$\mathbf{C_4H_8O_2}$
2	$\mathbf{C_4H_8O_2}$	$C_6H_{10}O_2$	$\mathbf{C_{13}H_{22}O}$	$\mathbf{C_{13}H_{22}O}$	$C_6H_{10}O_2$
3	$\mathbf{C_{13}H_{22}O}$	$C_{12}H_{24}O$	$C_{11}H_{22}O$	$C_{11}H_{22}O$	C_4H_8O
4	$C_{11}H_{22}O$	$C_4H_8O_2$	$C_2H_4O_2$	$C_6H_{10}O_2$	$C_{13}H_{22}O$
5	$C_2H_4O_2$	$C_{13}H_{22}O$	$C_{12}H_{24}O$	C_4H_8O	$C_2H_4O_2$
6	$C_6H_{10}O_2$	$C_7H_{14}O$	$C_9H_{16}O_2$	$C_7H_{14}O$	$C_{11}H_{22}O$
7	$C_7H_{14}O$	$C_{13}H_{26}O$	$C_7H_{14}O$	$C_9H_{16}O_2$	$C_7H_{14}O$
8	$C_9H_{16}O_2$	$C_6H_{12}O$	C_5H_8O	$C_2H_4O_2$	$C_6H_{12}O$
9	$C_6H_{12}O$	C_3H_4O	$C_{10}H_{20}O$	$C_{12}H_{24}O$	C_5H_8O
10	$C_{13}H_{26}O$	$C_2H_4O_2$	C_3H_4O	C_5H_8O	$C_{13}H_{26}O$
11	C_3H_4O	C_4H_8O	$C_{13}H_{26}O$	$C_6H_{12}O$	$C_9H_{16}O_2$
12	$C_8H_{16}O$	$C_8H_{16}O$	$C_8H_{16}O$	$C_{10}H_{20}O$	C_3H_4O
13	C_5H_8O	$C_{11}H_{22}O$	$C_6H_{12}O$	C_3H_4O	$C_8H_{16}O$
14		$C_9H_{16}O_2$	C_4H_8O	$C_8H_{16}O$	
15		$C_7H_{11}O$	$C_6H_{10}O_2$	$C_{13}H_{26}O$	

Table S4. Ranking of significant VOCs across clinical classifications. This table reproduces the ranking of the most important VOCs for binary classification as reported by Rai et al. (2022). Features are ranied based on their computed false discovery rate values. VOCs highlighted in bold represent the top three most significant features consistently identified in both our current analysis and the previous study. The classification scenarios include: Case I (Cancer vs. Control), Case II (Cancer vs. Benign), Case III (Benign vs. Control), Case IV (Cancer + Benign vs. Control), and Case V (Control + Benigh vs. Cancer).
