# Supplementary material for: Enhancing breath-based diagnostics through eXplainable Artificial Intelligence
Source: PLoS One. 2026 Jun 26;21(6):e0351833. doi: 10.1371/journal.pone.0351833 (PMC13308859; doi:10.1371/journal.pone.0351833)
Supplement: S5 Table — Data adapted from Rai et al (2022) to provide a reference for the feature profiles used in predictive modeling. (PDF) [file pone.0351833.s007.pdf]

Statistic	C_4H_8O	$C_6H_{12}O$	$C_7H_{14}O$	$C_8H_{16}O$	$C_{10}H_{20}O$	$C_{11}H_{22}O$	$C_{12}H_{24}O$	$C_{13}H_{26}O$	$C_4H_8O_2$	$C_2H_4O_2$	C_3H_4O	$C_6H_{10}O_2$	$C_9H_{16}O_2$	C_5H_8O	$C_7H_{11}O$	$C_{13}H_{22}O$
**Control (n = 193)**																
Mean	1.400	0.058	0.014	0.052	0.671	0.112	0.112	0.046	0.087	0.186	0.034	0.003	0.014	0.052	0.119	0.013
Median	1.370	0.021	0.007	0.025	0.516	0.089	0.078	0.012	0.073	0.181	0.000	0.001	0.000	0.000	0.000	0.000
SD	0.611	0.071	0.019	0.085	0.614	0.123	0.139	0.082	0.079	0.115	0.201	0.008	0.060	0.123	0.265	0.046
Min	0.200	0.000	0.000	0.000	0.007	0.000	0.000	0.000	0.000	0.001	0.000	0.000	0.000	0.000	0.000	0.000
Max	2.842	0.394	0.143	0.655	5.248	0.979	1.271	0.539	0.548	0.656	2.553	0.091	0.557	0.946	2.170	0.435
CV	0.44	1.22	1.41	1.61	0.91	1.10	1.25	1.77	0.91	0.62	5.88	2.55	4.36	2.36	2.23	3.44
SE	0.044	0.005	0.001	0.006	0.044	0.009	0.010	0.006	0.006	0.008	0.014	0.001	0.004	0.009	0.019	0.003
95%CI	(1.314, 1.486)	(0.048, 0.068)	(0.011, 0.016)	(0.041, 0.064)	(0.585, 0.758)	(0.094, 0.129)	(0.092, 0.131)	(0.035, 0.058)	(0.075, 0.098)	(0.17, 0.202)	(0.006, 0.063)	(0.002, 0.004)	(0.005, 0.022)	(0.035, 0.069)	(0.081, 0.156)	(0.007, 0.020)
**Benign Pulmonary Nodule (n = 65)**																
Mean	1.941	0.060	0.037	0.062	0.973	0.095	0.114	0.059	0.222	0.227	0.012	0.005	0.009	0.0675	0.1358	0.1568
Median	1.720	0.024	0.004	0.061	0.943	0.058	0.088	0.021	0.142	0.202	0.000	0.001	0.001	0.000	0.007	0.137
SD	0.930	0.117	0.134	0.047	0.682	0.141	0.151	0.125	0.277	0.163	0.044	0.008	0.022	0.151	0.333	0.131
Min	0.290	0.000	0.000	0.000	0.024	0.001	0.001	0.000	0.000	0.000	0.000	0.000	0.000	0	0	0
Max	4.570	0.841	1.016	0.216	3.308	0.854	0.929	0.739	1.830	0.765	0.315	0.053	0.102	0.723	1.832	0.393
CV	0.48	1.95	3.65	0.76	0.70	1.48	1.32	2.11	1.25	0.72	3.62	1.81	2.53	2.23	2.46	0.83
SE	0.115	0.014	0.017	0.006	0.085	0.017	0.019	0.015	0.034	0.020	0.005	0.001	0.003	0.019	0.041	0.016
95%CI	(1.715, 2.168)	(0.032, 0.088)	(0.004, 0.069)	(0.051, 0.074)	(0.807, 1.138)	(0.061, 0.129)	(0.078, 0.151)	(0.029, 0.089)	(0.155, 0.289)	(0.187, 0.267)	(0.001, 0.023)	(0.003, 0.007)	(0.003, 0.014)	(0.031, 0.104)	(0.055, 0.217)	(0.125, 0.189)
**Lung Cancer (n = 156)**																
Mean	3.273	0.147	0.041	0.076	1.033	0.111	0.146	0.064	0.449	0.368	0.014	0.016	0.007	0.045	0.143	0.161
Median	3.020	0.065	0.018	0.065	0.816	0.058	0.101	0.020	0.308	0.325	0.000	0.007	0.002	0.000	0.006	0.151
SD	1.454	0.268	0.069	0.102	1.172	0.269	0.264	0.207	0.423	0.270	0.051	0.033	0.022	0.183	0.513	0.134
Min	0.880	0.000	0.000	0.000	0.000	0.000	0.001	0.000	0.004	0.020	0.000	0.000	0.000	0	0	0
Max	8.210	2.122	0.658	0.730	11.933	2.808	2.808	2.150	2.539	2.110	0.469	0.325	0.249	1.691	4.314	0.750
CV	0.44	1.82	1.68	1.34	1.13	2.42	1.81	3.24	0.94	0.73	3.75	2.04	3.10	4.09	3.60	0.83
SE	0.116	0.021	0.006	0.008	0.094	0.022	0.021	0.017	0.034	0.022	0.004	0.003	0.002	0.015	0.041	0.011
95%CI	(3.045, 3.501)	(0.105, 0.189)	(0.030, 0.052)	(0.060, 0.092)	(0.849, 1.217)	(0.069, 0.153)	(0.104, 0.187)	(0.031, 0.097)	(0.383, 0.516)	(0.325, 0.410)	(0.006, 0.0022)	(0.011, 0.021)	(0.004, 0.011)	(0.016, 0.073)	(0.062, 0.223)	(0.140, 0.182)

Table S5. Summary statistics of the most relevant VOCs for the three clinical classes (Control, Benign, Cancer) in the Lung Cancer dataset. Data adapted from Rai et al (2022) to provide a reference for the feature profiles used in predictive modeling.
